# Dynamic finite-strain modelling of the human left ventricle in health and disease using an immersed boundary-finite element method

**DOI:** 10.1093/imamat/hxu029

**Published:** 2014-07-01

**Authors:** Hao Gao, David Carrick, Colin Berry, Boyce E. Griffith, Xiaoyu Luo

**Affiliations:** School of Mathematics and Statistics, University of Glasgow, Glasgow, UK; Institute of Cardiovascular and Medical Science, University of Glasgow, Glasgow, UK; Institute of Cardiovascular and Medical Science, University of Glasgow, Glasgow, UK; Leon H. Charney Division of Cardiology, Department of Medicine, New York University School of Medicine, New York, NY, USA and Department of Mathematics, Courant Institute of Mathematical Sciences, New York University, New York, NY, USA; School of Mathematics and Statistics, University of Glasgow, Glasgow, UK

**Keywords:** immersed boundary method, finite element method, left ventricle, magnetic resonance imaging, myocardial infarction, hyperelasticity, invariant-based constitutive model, excitation–contraction coupling

## Abstract

Detailed models of the biomechanics of the heart are important both for developing improved interventions for patients with heart disease and also for patient risk stratification and treatment planning. For instance, stress distributions in the heart affect cardiac remodelling, but such distributions are not presently accessible in patients. Biomechanical models of the heart offer detailed three-dimensional deformation, stress and strain fields that can supplement conventional clinical data. In this work, we introduce dynamic computational models of the human left ventricle (LV) that are derived from clinical imaging data obtained from a healthy subject and from a patient with a myocardial infarction (MI). Both models incorporate a detailed invariant-based orthotropic description of the passive elasticity of the ventricular myocardium along with a detailed biophysical model of active tension generation in the ventricular muscle. These constitutive models are employed within a dynamic simulation framework that accounts for the inertia of the ventricular muscle and the blood that is based on an immersed boundary (IB) method with a finite element description of the structural mechanics. The geometry of the models is based on data obtained non-invasively by cardiac magnetic resonance (CMR). CMR imaging data are also used to estimate the parameters of the passive and active constitutive models, which are determined so that the simulated end-diastolic and end-systolic volumes agree with the corresponding volumes determined from the CMR imaging studies. Using these models, we simulate LV dynamics from enddiastole to end-systole. The results of our simulations are shown to be in good agreement with subject-specific CMR-derived strain measurements and also with earlier clinical studies on human LV strain distributions.

## 1. Introduction

Cardiovascular diseases are the leading causes of death worldwide and account for ~17 million deaths annually, or ~30% of all deaths worldwide (WHO, 2011). Of these, >40% result from coronary heart disease. One of the most common outcomes of coronary heart disease is myocardial infarction (MI). Following an MI, appropriate measures must be taken quickly to prevent the development of chronic heart failure. Untreated or unsuccessfully treated MIs can cause extensive fibrous scarring and the expansion of the infarct region, hypertrophy of the remote myocardium and ventricular dilatation. These adverse remodelling processes can impair cardiac pump function and can lead to lethal arrhythmias such as ventricular fibrillation. The processes underlying heart function and dysfunction are complex, and there is growing recognition that it is not possible to identify all of the key mechanisms of the disease by experimental approaches alone. Hence, there is a clear need for integrative mathematical and computational models of the heart that can help to understand the interplay of physiological and pathophysiological mechanisms at the molecular, cellular, tissue and organ scales in both the ischaemic region and in the remote healthy myocardium.

A common approach to modelling the passive elastic response of the ventricular myocardium is to use a transversely isotropic hyperelastic strain-energy function (Guccione *et al.*, 1991; Bovendeerd *et al.*, 1992, 2009; Wenk *et al.*, 2012). The increasing availability of detailed experimental data (Dokos *et al.*, 2002) has led to the development of orthotropic constitutive models that more completely describe the laminar architecture of the ventricular myocardium (Nash & Hunter, 2000; Costa *et al.*, 2001; Holzapfel & Ogden, 2009). Usyk *et al.* (2000) demonstrated that orthotropic models yield superior agreement with measured end-diastolic strains than transversely isotropic models, although this effect is reduced at end-systole. Bovendeerd *et al.* (2009) suggested that more accurate shear strains can be obtained by incorporating the transmural crossover of myofibres in the constitutive model. Aguado-Sierra *et al.* (2011) also found that an orthotropic model yields better agreement with experimentally characterized pressure–volume relationships than a transversely isotropic model; specifically, the orthotropic model is better able to capture the power law-like behaviour of the pressure–volume relationship. Recently, using the invariant-based orthotropic model of Holzapfel & Ogden (2009), Wang et al. developed a medical image-based left ventricle (LV) model and explored the effects of the fibre and sheet angle distributions (Wang *et al.*, 2013a) and residual stress (Wang *et al.*, 2014) on the diastolic mechanics of the LV. A more complete review of models of the passive elasticity of the myocardium is provided by Holzapfel & Ogden (2009).

Determining the entire set of constitutive parameters of detailed models of ventricular mechanics using *in vivo* data is challenging. One approach is to use parameters obtained from animal studies as initial estimates, and then to adjust those parameters to fit *in vivo* measurements such as displacements, strains or pressure–volume relationships (Walker *et al.*, 2005; Sun *et al.*, 2009; Wang *et al.*, 2009; Nordsletten *et al.*, 2010; Aguado-Sierra *et al.*, 2011; Wenk *et al.*, 2012). Wenk *et al.* (2012) used the transversely isotropic constitutive model of Guccione *et al.* (1991) and determined one passive and one active material parameter while leaving the remaining parameters fixed. Sun *et al.* (2009) employed a gradient-free approach to determine regional contractility parameters. A similar approach was adopted by Nordsletten *et al.* (2010), who adjusted a single proportionality coefficient that was applied to all of the model parameters. Xi *et al.* (2011a) also used a constitutive model based on a transversely isotropic model of Guccione *et al.* (1991) and proposed a reduced-order unscented Kalman filter to estimate four passive material parameters for synthetic LV motions.

To predict the state of stress in both diastole and systole, it is also necessary to model active tension generation. Active models of the heart that incorporate transmural variations in fibre orientation and non-linear hyperelastic passive responses have been developed by various groups, including Bovendeerd *et al.* (1992), Huyghe *et al.* (1992) and Guccione *et al.* (1995). Nash & Hunter (2000) developed a finite element (FE) framework for large-deformation heart simulation using the ‘pole-zero’ constitutive law and a simplified model for active contraction to predict myocardial strain at end-systole. Usyk *et al.* (2000) considered the effects of active stress with and without active transverse components, and they found that systolic shear strains are more accurately predicted when transverse components are included in the description of active stress. Kerckhoffs *et al.* (2007) developed a multiscale model of the canine ventricles that was coupled to lumped systemic and pulmonary circulation models, thereby enabling realistic multibeat simulations. More advanced models that include descriptions of activation that are based on the monodomain or bidomain models of electrical excitation propagation have also been developed (Aguado-Sierra *et al.*, 2011; Eriksson *et al.*, 2013). Such models may be used to investigate the effects of ventricular pacing on cardiac function (Kerckhoffs *et al.*, 2009; Niederer *et al.*, 2011b; Kuijpers *et al.*, 2012).

Various diseased heart models have also been developed. Using a computational model of the ovine LV with an anteroapical transmural MI, Guccione *et al.* (2001) proposed that the mechanical dysfunction in the ‘border zone’ between the healthy and infarcted regions of the myocardium results from contractile dysfunction rather than altered wall stresses. To further investigate the mechanism underlying the dysfunction of the infarct border zone, they developed systolic computational LV models and optimized both the active and passive material parameters by matching the diastolic and systolic LV cavity volumes and strains with clinical measurements (Walker *et al.*, 2005; Sun *et al.*, 2009). In a series of diseased animal and human LV models, Guccione and colleagues found that the myocardial contractility in the infarct border zone is substantially reduced, but with an elevated myofibre stress, which may lead to adverse remodelling and progressive heart failure (Wenk *et al.*, 2011a,b, 2012; Zhang *et al.*, 2012).

In contrast to the vast literature on modelling the structural mechanics of the heart, relatively few studies account for intracardiac fluid dynamics and fluid–structure interaction (FSI). It seems likely that this is a consequence of the additional computational challenges posed by such models, including but not limited to solving the Navier–Stokes equations. Several groups have developed FSI models of the LV using the arbitrary Lagrangian–Eulerian (ALE) approach. For instance, Watanabe *et al.* (2004) coupled FSI and electrophysiological dynamics in a LV model. Nordsletten *et al.* (2010) developed a non-conforming ALE framework for simulating blood flow and myocardial dynamics in both the diastolic and systolic phases of the cardiac cycle that coupled the fluid and solid via a Lagrange multiplier field defined on the fluid–solid interface, and McCormick *et al.* (2011) used this model to investigate the impact of a left-ventricular assist device on overall LV pump function.

The ALE approach is a natural extension of the FE method to FSI problems, but such schemes may yield significant computational difficulties due to dynamic mesh generation caused by large structural deformations. The immersed boundary (IB) method (Peskin, 2002) is an alternative approach to FSI that was introduced by Peskin to simulate the fluid dynamics of heart valves (Peskin, 1972), and that was subsequently extended by Peskin and McQueen to simulate the dynamics of the heart, its valves and the nearby great vessels (Peskin & McQueen, 1996; McQueen & Peskin, 1997, 2000; Kovács *et al.*, 2001; McQueen & Peskin, 2001; Griffith *et al.*, 2007, 2009a). The IB approach to FSI treats the particular case in which an incompressible structure is immersed in a viscous incompressible fluid, using a Lagrangian description of the deformations and stresses of the immersed structure and an Eulerian description of the momentum, viscosity and incompressibility of the coupled fluid–structure system. Interaction between Lagrangian and Eulerian variables is mediated by integral transforms with Dirac delta function kernels. When the continuous equations are discretized, the Eulerian equations are approximated on a Cartesian grid and the Lagrangian equations are approximated on a curvilinear mesh. A key feature of the IB method is that it does not require dynamically generated body-conforming discretizations of the fluid and solid. Instead, the Lagrangian mesh overlays the Eulerian grid, and interplay between Lagrangian and Eulerian quantities occurs via discretizations of the integral transforms involving regularized delta function kernels. The IB method thereby effectively circumvents the mesh distortion problems of ALE methods for systems involving large structural deformations.

In this study, we develop dynamic models of LV function in health and disease using a version of the IB method (Griffith & Luo) that uses Lagrangian FE methods to describe the passive and active response of the ventricular myocardium. The geometry and regional function of the LV are reconstructed from clinical images acquired from a healthy subject and from a patient 6-months following an acute MI. The passive elasticity of the LV is described using the invariant-based orthotropic constitutive model of Holzapfel & Ogden (2009), and excitation–contraction coupling and active tension generation are described using the model of Niederer *et al.* (2006). In the MI model, we include heterogeneous material properties that account for the functional differences in the unaffected myocardium, the infarcted region and the infarct border zone. Passive and active material parameters are empirically determined so that the predicted end-diastolic and end-systolic volumes agree with image-based measurements of left ventricular volume (Sun *et al.*, 2009). Because the models lack descriptions of the cardiac valves, their ability to predict realistic flow patterns is limited, but the IB framework does enable us to account for the inertia of both the left ventricular wall and also the blood. Nonetheless, these models enable us to quantify regional stresses and strains throughout the cardiac cycle. Our results are shown to be in good agreement with subject-specific strains derived from cardiac magnetic resonance (CMR) images as well as earlier studies on strain distributions in the human heart.

## 2. Methodology

### 2.1 IB formulation

The IB method (Peskin, 2002) models systems in which an incompressible solid is immersed in a viscous incompressible fluid. In the IB formulation of such FSI problems, the deformations and stresses of the immersed solid are described using Lagrangian variables, and the momentum, viscosity and incompressibility of the fluid–solid system are described using Eulerian variables. Let $\Omega \subset R^{3}$ denote the physical domain occupied by the fluid–solid system, and let $U\subset R^{3}$ denote the reference coordinate system attached to the immersed solid. We use **x** = (*x*_1_, *x*_2_, *x*_3_) ∈ *Ω* to indicate fixed physical (Eulerian) coordinates and **X** = (*X_1_*, *X_2_*, *X*_3_ ∈ *U* indicate material (Lagrangian) coordinates attached to the immersed solid. The present physical position of material point **X** at time *t* is ***χ***(**X**, *t*) ∈ *Ω*, the physical region occupied by the immersed structure at time *t* is ***χ***(*U, t*) *Ω*^s^(*t*) ⊂ *Ω* and the physical region occupied by the fluid at time *t* is *Ω*^f^(*t*) = *Ω* \ *Ω*^s^(*t*). In our models, *U* = *Ω*^s^(*t*_0_) at the initial time *t* = *t*_0_, although this is not a requirement of either the mathematical formulation or our implementation.

The continuous equations of motion for the coupled fluid–structure system are (Boffi *et al.*, 2008)

(2.1) $$\rho \left(\frac{\partial u}{\partial t}\left(x,t\right)+u\left(x,t\right)\cdot \nabla u\left(x,t\right)\right)=-\nabla p\left(x,t\right)+\mu \nabla^{2}u\left(x,t\right)+f^{s}\left(x,t\right),$$

(2.2) ∇⋅u(x,t)=0,

(2.3) $$f^{s}\left(x,t\right)=\int_{U}\nabla_{X}\cdot P^{s}\left(X,t\right)\;\delta \left(x-\chi \left(X,t\right)\right)\;dX-\int_{\partial U}P^{s}\left(X,t\right)\;N\left(X\right)\;\delta \left(x-\chi \left(X,t\right)\right)\;dA,$$

(2.4) $$\frac{\partial \chi }{\partial t}\left(X,t\right)=\int_{\Omega }u\left(x,t\right)\;\delta \left(x-\chi \left(X,t\right)\right)\;dx,$$

in which *ρ* is the mass density of the fluid–structure system, *μ* is the viscosity, **u**(**x**, *t*) is the Eulerian velocity field of the fluid–structure system, *p*(**x**, *t*) is the Eulerian pressure field of the fluid–structure system, **f**^s^(**x**, *t*) is the Eulerian force density generated by the passive elasticity and the active contraction of the immersed solid, $P^{s}$(**X**, *t*) is the First Piola–Kirchhoff stress tensor associated with the passive elasticity and active contraction of the immersed solid, **N**(**X**) is the exterior unit normal to *U* and *δ*(**x**) = *δ*(*x*_1_) *δ*(*x*_2_) *δ*(*x*_3_) is the three-dimensional Dirac delta function.

This IB formulation (2.1–2.4) has two Lagrangian–Eulerian interaction equations involving integral transforms with delta function kernels. The first interaction equation (2.3) converts a Lagrangian description of the structural stresses into an equivalent Eulerian body force **f**^s^. To understand the role of **f**^s^ and $P^{s}$ in determining the total stress of the coupled fluid–structure system, we remark that it is possible to express the right-hand side of momentum equation (2.1) as (Boffi *et al.*, 2008)

(2.5) $$-\nabla p\left(x,t\right)+\mu \nabla^{2}u\left(x,t\right)+f^{s}\left(x,t\right)=\nabla \cdot \sigma \left(x,t\right),$$

in which **σ**(**x**, *t*) is the Cauchy stress tensor of the coupled fluid–structure system. Further, it can be shown that in this formulation,

(2.6) σ(x,t)=−pI+μ[∇u+(∇u)T]+{σs(x,t)forx∈Ωs(t),0forx∈Ωf(t),}

in which $-pI+\mu \left[\nabla u+{\left(\nabla u\right)}^{T}\right]$ is the fluid-like stress existing in both *Ω*^f^ and *Ω*^s^, and ***σ***^s^(**x**, *t*) describes the passive elastic and active contractile stresses of the LV. The Cauchy stress ***σ***^s^ in equation (2.6) is related to the first Piola–Kirchhoff stress tensor $P^{s}$ via

(2.7) $$\sigma^{s}=\frac{1}{J}P^{s}F^{T},$$

in which F=∂χ∕∂X is the deformation gradient tensor associated with the mapping χ:(U,t)↦Ω and J=det(F). Although we refer to ***σ***^s^ and $P^{s}$ as the structural stresses, it is important to keep in mind that ***σ***^s^ and $P^{s}$ are not the total stress of the immersed solid. Rather, ***σ***^s^ and $P^{s}$ only account for the stresses associated with the hyperelastic and active material responses. The total stress of the immersed solid is $\sigma =-pI+\mu \left[\nabla u+{\left(\nabla u\right)}^{T}\right]+\sigma^{s}$; see equation (2.6).

The second Lagrangian–Eulerian interaction equation (2.4) specifies the velocity of the immersed solid in terms of the Eulerian velocity field. Because of the presence of viscosity, **u**(**x**, *t*) is a continuous velocity field. Hence, equation (2.4) implies

(2.8) $$\frac{\partial \chi }{\partial t}\left(X,t\right)=u\left(\chi \left(X,t\right),t\right),$$

so that **u**(**x**, *t*) is the Eulerian description of the velocity of the coupled fluid–solid system. Specifically, if **x** ∈ *Ω*^f^(*t*) at time *t*, then **u**(**x**, *t*) is the velocity of the fluid located at position **x** at time *t*, and if **x** *Ω*^s^(*t*) at time *t*, then **u**(**x**, *t*) is the velocity of the solid at that position. Notice that equation (2.8) implies that if ∇ · **u** = 0 then *∂J/∂t* = 0. Consequently, if *J* = 1 at *t* = *t*_0_, then *J* ≡ 1 for all *t* ≥ *t*_0_.

We remark that the formulation 2.1–2.4) assumes that the mass density and viscosity of the fluid and solid are equal. Although these assumptions simplify the implementation, they are not essential, and versions of the IB method have been developed that permit the use of spatially varying structural mass densities (Zhu & Peskin, 2002; Kim *et al.*, 2003; Kim & Peskin, 2007; Mori & Peskin, 2008; Roy *et al.*, 2013) and viscosities (Fai *et al.*, 2013; Roy *et al.*, 2013). In the simulations detailed herein, we set *ρ* = 1.0 g/ml and *μ* 0.04 cP.

In practice, we discretize these equations of motion using a finite difference (FD) method for the Eulerian equations and a FE method for the Lagrangian equations. To use standard Lagrangian FE methods, it is useful to introduce an equivalent weak formulation of the definition of **f**^s^ in equation (2.3), namely

(2.9) $$f^{s}\left(x,t\right)=\int_{U}F^{s}\left(X,t\right)\delta \left(x-\chi \left(X,t\right)\right)\;dX,$$

(2.10) $$\int_{U}F^{s}\left(X,t\right)\cdot V\left(X\right)\;dX=-\int_{U}P^{s}\left(X,t\right):\nabla_{X}V\left(X\right)\;dX,$$

in which **F**^s^(**X**, *t*) is the Lagrangian elastic force density and **V**(**X**) is an arbitrary Lagrangian test function that is not assumed to vanish on *∂U*. Although equations (2.3) and (2.9–2.10) are equivalent in the continuous setting, they lead to different numerical schemes, and equations (2.9–2.10) allow **F**^s^(**X**, *t*) to be determined via a standard total Lagrangian FE scheme. Further details are provided by Griffith & Luo.

### 2.2 Left ventricular anatomy and function

To acquire data on LV anatomy and functional status, CMR images were acquired from a healthy volunteer (male, age 28) at the British Heart Foundation Cardiovascular Research Center at the University of Glasgow using a Siemens (Erlangen, Germany) Magnetom Verio 3 T scanner, and from a patient 6 months after an acute MI (female, age 41) at Golden Jubilee National Hospital using a Siemens Magnetom Avanto 1.5 T scanner. The studies were approved by the ethics committee at the University of Glasgow, and written informed consent was obtained prior to the studies. In both cases, the basic CMR imaging protocol was as follows: steady-state free precession cine imaging was used for functional assessment, and a short-axis cine stack of the LV from the base to the apex was acquired that consisted of 7-mm thick slices with a 3-mm interslice gap. Cine images were also obtained in the three-chamber, horizontal long-axis and vertical long-axis planes. To identify the infarcted region of the patient's LV, an additional late gadolinium enhancement (LGE) sequence was performed in the patient study.

**Fig. 1:**
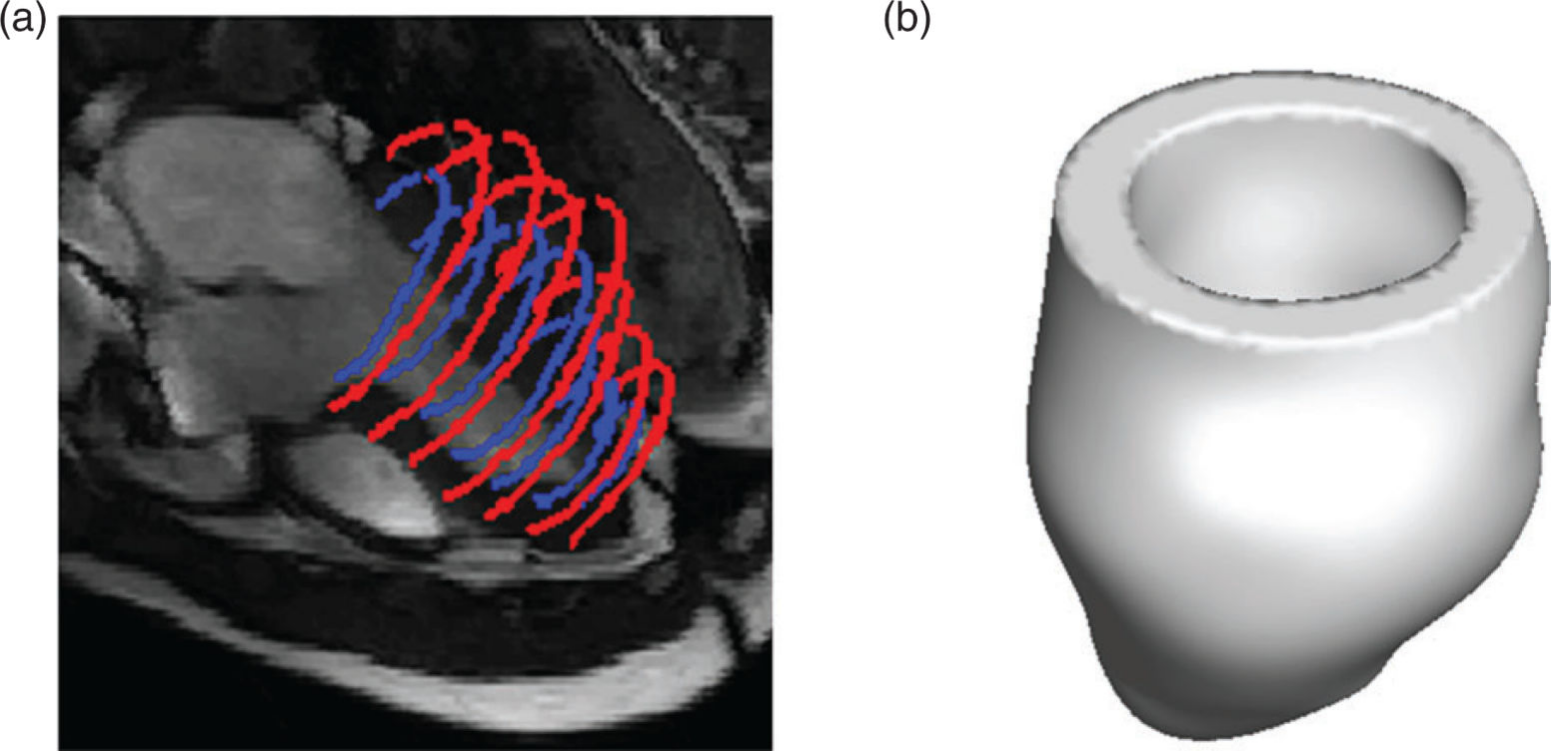
LV geometry reconstruction for the healthy volunteer: (a) the endocardial (blue online) and epicardial (red online) boundary segmentations and (b) the reconstructed healthy LV model.

For each data set, the custom MATLAB (Mathworks, Inc., Natick, MA, USA) software was used to extract the endocardial and epicardial surfaces of the LV at early diastole. Seven short-axis slices from the base to the apex (matrix size: 216 × 256 for the healthy subject and 180 × 256 for the patient; slice distance: 10 mm) and three long-axis slices were selected for segmentation. To determine the LV geometry, SolidWorks (Dassault Systèmes SolidWorks Corp., Waltham, MA, USA) was used to obtain B-spline surface fits of the segmentation data. Figure 1 shows the LV segmentation and reconstruction for the healthy subject.

**Fig. 2:**
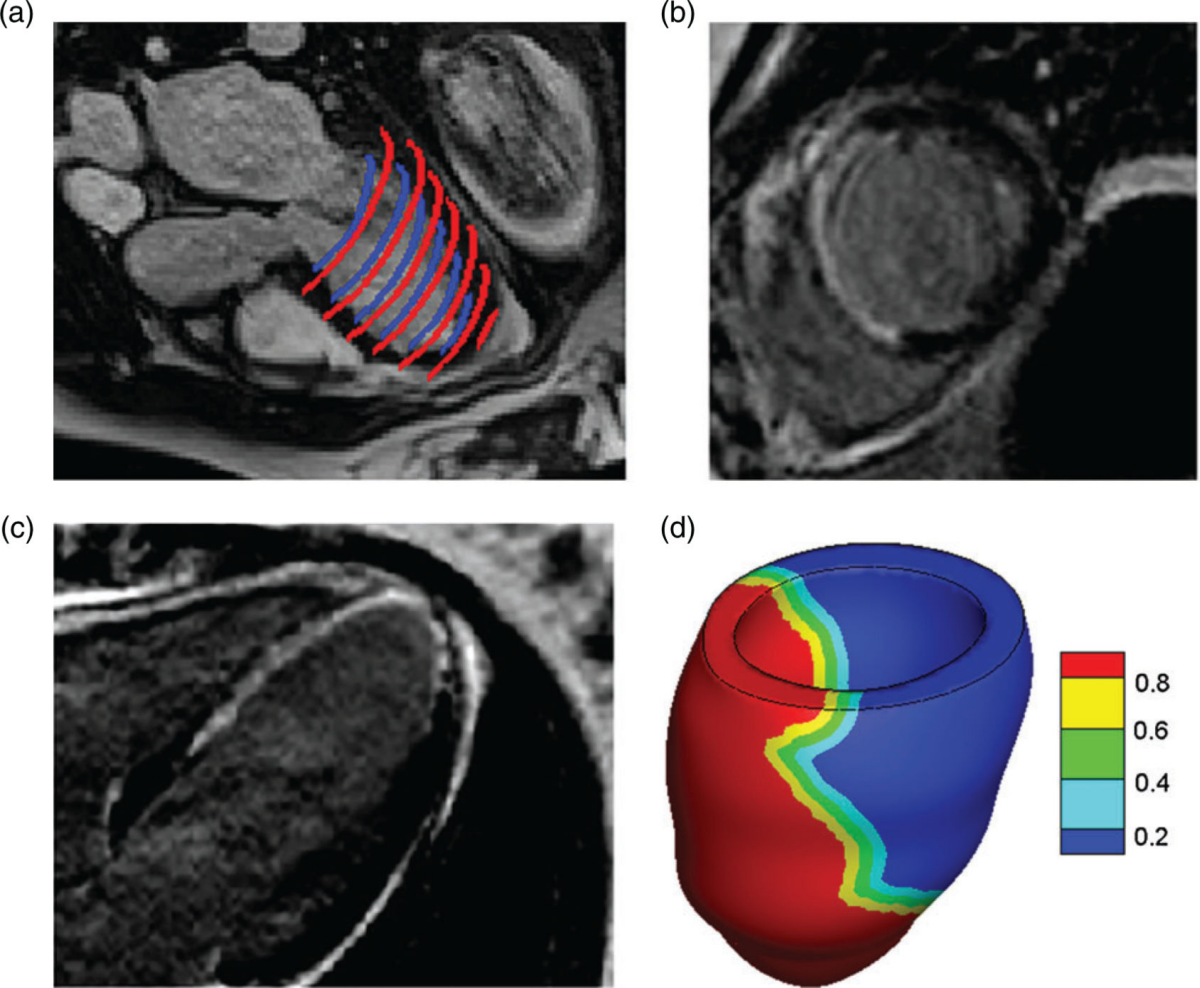
LV geometry reconstruction for the MI patient: (a) endocardial (blue online) and epicardial (red online) boundary segmentations; (b) short-axis LGE MR image slice (the enhanced bright region indicates MI); (c) long-axis LGE MR image slice and (d) reconstructed LV model contoured by the LGE image-based model of MI extent (0: unaffected healthy region; 1: reconstructed infarcted region). A 10-mm thick transition region (border zone) is assumed to lie between the unaffected (blue or right) and infarct (red or left) regions.

For the patient, short- and long-axis LGE images were combined with the cine MR images to reconstruct the infarcted region, which is shown in Fig. 2. The reconstructed infarct region, denoted *U*^in^, is 60% of the total volume of the reconstructed LV region *U* and is located mainly in the septal and apical regions of the LV; see Fig. 2(b,c). The extent of the transition region (i.e. border zone) cannot be extracted from the images. To model the effects of the transition region, we defined the border zone region, denoted *U*^bz^, to include all points exterior to *U*^in^ within a distance $\ell^{\mathrm{bz}}=10\;\mathrm{mm}$ of the boundary of the infarcted region as in prior studies by Nordsletten *et al.* (2010). The remainder of the LV volume was assumed to be unaffected by the infarct and is denoted *U*^un^. To account for the heterogeneous nature of the infarct border zone, a Lagrangian field *M*(**X**) indicating the extent of the infarction was defined throughout the tissue volume *U* so that

(2.11) M(X)={1ifX∈Uin,1−dun(X)ℓbzifX∈Ubzand0ifX∈Uun,}

in which

(2.12) $$d^{\mathrm{un}}\left(X\right)=\min_{X^{\prime }\in U^{\mathrm{in}}}\left\|X-X^{\prime }\right\|.$$

By construction, *M* is continuous and takes the value 1 on *U*^in^ and the value 0 on *U*^un^ [see Fig. 2(d)].

Because high-resolution non-invasive methods for determining the *in vivo* fibre architecture of the heart are not routinely available, a rule-based fibre generation method based on the work of Bishop *et al.* (2009); Potse *et al.* (2006) was used to construct the fibre architecture of the myocardium, as we have done in earlier studies (Wang *et al.*, 2013a, 2014; Gao *et al.*, 2014b). In this construction, the fibre angle was assumed to rotate from −60° to 60° from the endocardium to the epicardium, and the sheet angle was assumed to rotate from −45° to 45°.

**Fig. 3:**
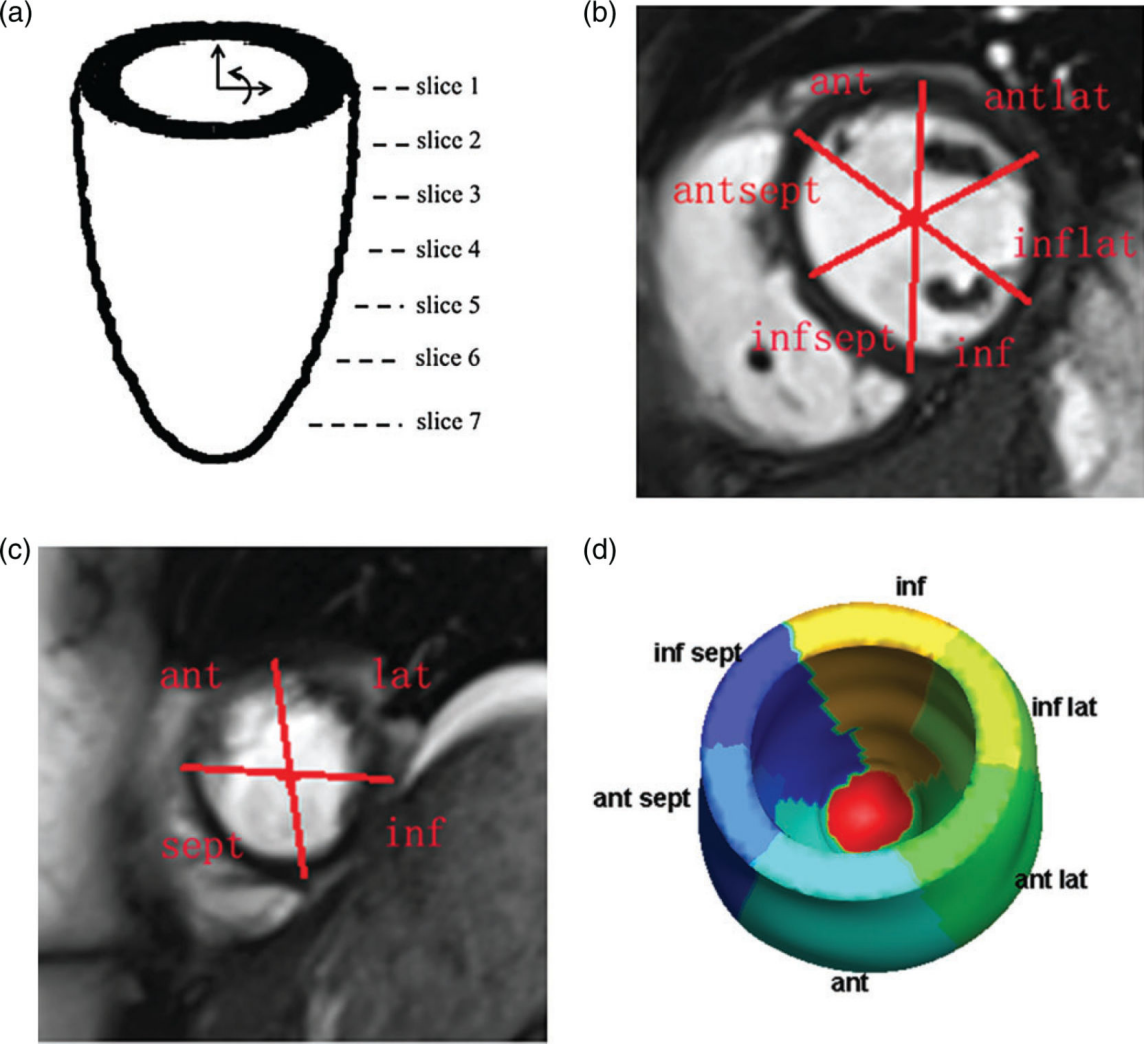
The LV is divided into regions on selected short-axis slices: (a) the positions of the seven short-axis slices; (b) the regions defined on slices 1–5; (c) the regions defined on slices 6 and 7 and (d) the corresponding divisions of the LV wall.

The LV was divided into regions corresponding to seven short-axis CMR slices, starting from the basal plane, as shown in Fig. 3(a). The first five slices were divided into six segments: inferior septal (infsept), anterior septal (antsept), anterior (ant), anterior lateral (antlat), inferior lateral (inflat) and inferior (inf), as shown in Fig. 3(b). The two slices near the apex are smaller and hence were divided into only four regions: septal (sept), anterior (ant), lateral (lat) and inferior (inf), as shown in Fig. 3(c). Note that these regions are defined only on a discrete collection of planes. To associate a unique region with a generic point **X** ∈ *U*, **X** was orthogonally projected onto the nearest slice plane [see Fig. 3(d)].

### 2.3 Myocardial mechanics

We model the first Piola–Kirchhoff myocardial stresses that account for the passive elasticity and active tension generation in the LV as

(2.13) $$P^{s}=P^{p}+P^{a},$$

in which $P^{p}$ corresponds to the passive elastic response and $P^{a}$ is the active stress. Recall that the total Cauchy stress ***σ*** within the immersed solid is given by equation (2.6), and that $P^{s}$ accounts for the hyperelastic and active material responses but not for the incompressibility of the immersed solid.

The orthotropic passive elastic response of the myocardium is described by an invariant-based hyperelastic energy functional *W* introduced by Holzapfel & Ogden (2009). For the healthy model, this is

(2.14) $$W=\frac{a}{2b}\exp\left[b\left(I_{1}-3\right)\right]+\sum_{i=f,s}\frac{a_{i}}{2b_{i}}\left\{\exp\left[b_{i}{\left(I_{4i}^{⋆}-1\right)}^{2}\right]-1\right\}+\frac{a_{\mathrm{fs}}}{2b_{\mathrm{fs}}}\left\{\exp\left[b_{\mathrm{fs}}{\left(I_{8\mathrm{fs}}\right)}^{2}\right]-1\right\},$$

in which $I_{1}=\mathrm{tr}\left(C\right)$ is the first invariant of the right Cauchy–Green deformation tensor $C=F^{T}F$ and $I_{4f}^{⋆}$, $I_{4s}^{⋆}$ and *I*_8fs_ are invariants that account for the passive anisotropic and shear properties of the myocardium. Denoting the unit fibre and sheet axes in the reference configuration by **f**_0_ = **f**_0_(**X**) and **s**_0_ = **s**_0_(**X**), respectively, $I_{4f}^{⋆}$ and $I_{4s}^{⋆}$ are defined in terms of

(2.15) $$I_{4f}=f_{0}^{T}Cf_{0}=f\cdot f\;\text{and}\;I_{4s}=s_{0}^{T}Cs_{0}=s\cdot s,$$

in which $f=Ff_{0}$ and $s=Fs_{0}$ are the fibre and sheet axes in the current configuration. Note that *I*_4f_ and *I*_4s_ are the squares of the stretches in the fibre and sheet directions, *λ*_f_ and *λ*_s_, respectively. The modified invariants $I_{4i}^{⋆}$ are defined in terms of *I*_4*i*_ by

(2.16) $$I_{4i}^{⋆}=\max\left(I_{4i},1\right)$$

for *i* = f or s. This ensures that the elastic energies associated with load-bearing collagen fibres embedded in the myocardium are non-zero only when the fibres are in states of extension (Holzapfel & Ogden, 2009). *I*_8fs_ is defined by

(2.17) $$I_{8\mathrm{fs}}=f_{0}^{T}Cs_{0}=f\cdot s.$$

This last invariant is needed to account fully for the shear properties of the myocardium (Holzapfel & Ogden, 2009).

The Lagrangian First Piola–Kirchhoff passive elastic stress tensor $P^{p}$ is defined in terms of *W* by

(2.18) $$P^{p}=\frac{\partial W}{\partial F}-a\;\exp\left[b\left(I_{1}-3\right)\right]F^{-T},$$

so that

(2.19) $$\sigma^{p}=\frac{1}{J}\frac{\partial W}{\partial F}F^{T}-p^{s}I.$$

where *p*^s^ = (1/*J*)*a* exp[*b*(*I*_1_ – 3)]. Note that in our formulation, the pressure-like term *p*^s^ ensures that for $F=I,P^{p}=\sigma^{p}=0$, and *p*(**x**, *t*) is constant. *p*^s^ does not enforce the incompressibility constraint *J* = 1, which is implicitly satisfied by the constraint ∇ · **u** 0; see equation (2.8). In the continuous equations, it is not necessary to impose the incompressibility constraint in the Lagrangian form. In the discretized equations, however, we have demonstrated it can be important also to impose the incompressibility constraint in the Lagrangian form (Gao *et al.*, 2014b), because in the spatially discrete equations, the Lagrangian structural velocity field is not guaranteed to be divergence free (either discretely or continuously). This lack of incompressibility has a relatively minor effect on the computed structural displacements, but it can have a large effect on the computed stresses. In our earlier work (Gao *et al.*, 2014b), we found that we recover the structural stresses more accurately by including a penalty term to reinforce the incompressibility constraint in the Lagrangian form. With this additional constraint, the Piola–Kirchhoff stress becomes

(2.20) $${\tilde{P}}^{p}=\frac{\partial W}{\partial F}+\left\{-a\;\exp\left[b\left(I_{1}-3\right)\right]+\beta_{s}\;\log\left(I_{3}\right)\right\}F^{-T},$$

in which $I_{3}=\det\left(C\right)=J^{2}$, and *β_s_* = 1.0e6 dyne/cm^2^. The physical pressure is now *p + p*^s^ – *β_s_*(log(*J*^2^)/*J*). Although linear tetrahedral elements will yield volumetric locking for sufficiently large values of *β_s_*, comparisons to a simplified geometrical model constructed using both hexahedral and tetrahedral elements verify that for the value of *β_s_* used in this study, our model does not experience volumetric locking (data not shown).

The first Piola–Kirchhoff active stress tensor $P^{a}$ is defined by

(2.21) $$P^{a}=JTFf_{0}⊗f_{0},$$

in which *T*(**X**, *t*) is the active tension. Recall that **f**_0_ is the normalized fibre direction field in the reference configuration. The corresponding Cauchy stress is

(2.22) $$\sigma^{a}=Tf⊗f.$$

In this work, *T* is determined by the active contraction model of Niederer *et al.* (2006), which determines the active contraction as a function of the fibre stretch *λ*_f_, the time rate of change of the fibre stretch *∂λ*_f_/*∂t*, and the intracellular calcium concentration [Ca]_i_. Here, we use an analytically prescribed, spatially uniform model of intracellular calcium dynamics (Hunter *et al.*, 1998) to stimulate active contraction. The equations governing the dynamics of *T* are briefly summarized in the Appendix A. In our simulations, we control the strength of the active contractile force by optimizing the parameter *T*_scale_ defined therein so as to yield realistic end-systolic volumes. We do not determine the values of all of the model parameters due to the limited data available for use in parameter estimation (Krishnamurthy *et al.*, 2013; Sun *et al.*, 2009).

In the diseased LV model, the passive and active material responses both should differ in the unaffected region *U*^un^, the transition region *U*^bz^ and the infarct region *U*^in^. Recall that *M* is a continuous piecewise-linear function that models the local extent of the tissue damage resulting from the infarct, with *M* = 0 for **X** ∈ *U*^un^ and *M* = 1 for **X** ∈ *U*^in^. We assume that the passive response in the infarcted region is 50 times stiffer than that in the unaffected region as done by Wenk *et al.* (2011a), so that for the MI model, the strain energy functional is

(2.23) $$W^{\mathrm{MI}}=\left(1+49M\right)W.$$

Different material parameters are used for the healthy and MI models, as detailed in Sections 2.6 and 3.1. In the MI model, all heterogeneities in the passive material response are accounted for by the pre-factor (1 + 49*M*) in equation (2.23). Specifically, the same passive constitutive model parameters (i.e. *a*, *b*, *a_i_* and *b_i_* for *i* = f, s and fs) are used in the infarct, the border zone, and the healthy region of the LV. We also assume that in the infarct region, the tissue is non-contractile, so that in the diseased model

(2.24) $$T^{MI}=\left(1-M\right)T.$$

Note that by construction, *T*^MI^ continuously transitions to zero as **X** → *U*^in^. We also determine different values of *T*_scale_ for the healthy and MI models.

### 2.4 Boundary, loading and driving conditions

**Fig. 4:**
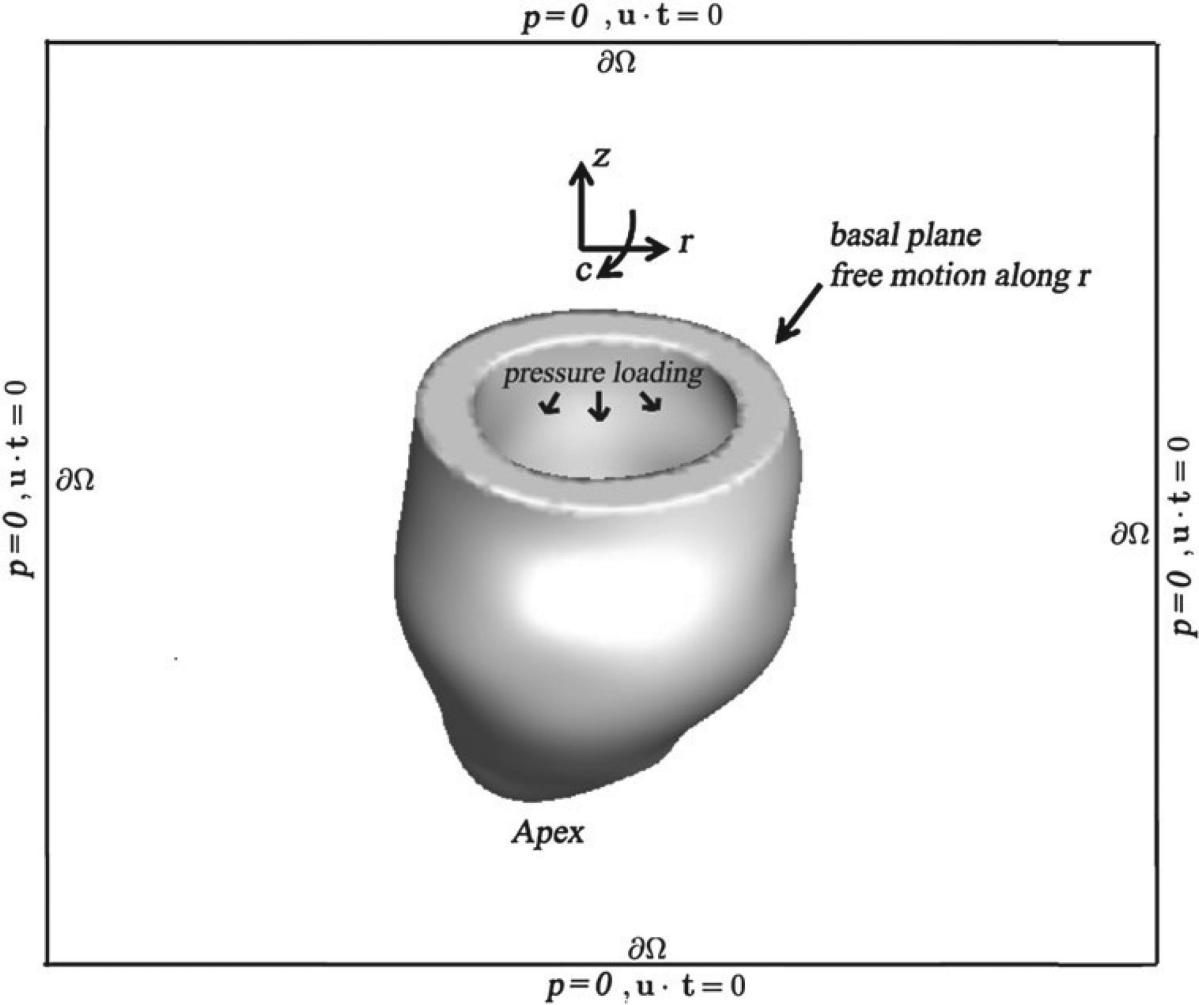
Illustration of boundary and loading conditions applied to the IB/FE LV models, adapted from Gao *et al.* (2014b). *c*: circumferential direction, *r*: radial direction, *z*: axial direction. LV cavity pressure loading is applied to the endocardial surface, and displacements of the basal plane are fixed in the *c* and *z* directions, therefore permitting only radial expansion. The whole computational domain is represented by the black box with zero pressure and zero tangential slip along *∂Ω*, where **u** is the Eulerian velocity and **t** is the unit tangential vector.

To constrain the motion of the LV, a penalty method approximately imposes zero axial and circumferential displacements along the basal plane. Radial displacements are not penalized, and the reminder of the left ventricular wall, including the apex, is also left free. Along *∂Ω*, we impose a combination of zero normal traction and zero tangential velocity boundary conditions. For an incompressible fluid, these boundary conditions imply that the pressure is zero along *∂Ω*. In our simulations, boundary conditions on *∂Ω* are imposed using an efficient multigrid-pre-conditioned Krylov method (Griffith, 2009, 2012). Figure 4 shows a schematic of the boundary and loading conditions.

In the dynamic simulations, a time-dependent but spatially uniform pressure load is applied to the endocardial portion of *∂Ω*^s^(*t*) (Krishnamurthy *et al.*, 2013; Wang *et al.*, 2013a; Wenk *et al.*, 2011a). This additional pressure loading appears as an additional boundary term in the definition of the Lagrangian force density **F**^s^, as in a standard Lagrangian FE method, and is implemented in a purely Lagrangian fashion. We first increase the endocardial pressure to an assumed end-diastolic value and allow the model to achieve its measured end-diastolic volume. The pressure is then rapidly increased to the endsystolic value, which is approximated by the measured brachial arterial pressure. The intracellular calcium concentration is simultaneously increased to its peak value to induce active tension (Wenk *et al.*, 2011a). The computation ends when a steady state is reached.

### 2.5 Discretization and implementation

In our simulations, *Ω* is taken to be a 15 cm × 15 cm × 20 cm box that is discretized with grid spacings *Δx* = *Δx*_1_ = *Δx*_2_ = *Δx*_3_ = 0.156 cm, corresponding to a regular 96 × 96 × 128 Cartesian grid. In the numerical scheme, the singular delta function kernel appearing in the Lagrangian–Eulerian interaction equations (2.4) and (2.9) is replaced by a standard four-point regularized version of the delta function (Peskin, 2002). The integral transforms appearing in the interaction equations are approximated using dynamically generated Gaussian quadrature rules that ensure a density of at least two quadrature points per Cartesian mesh width. Further details of the spatial discretizations and time stepping scheme are provided by Griffith & Luo. A time step size of 1.22e–4 s is used in diastole, and a time step size (3.0e–5 s) is used in systole. The small time step size is required because of the explicit time stepping scheme employed by our implementation (Griffith & Luo).

All simulations of active contraction use the open-source IBAMR software (IBA; Griffith *et al.*, 2007, 2009a), which is an adaptive and distributed-memory parallel implementation of the IB method. IBAMR uses other open-source libraries, including SAMRAI (SAMRAI; Hornung & Kohn, 2002), PETSc (Balay *et al.*, 2013a,b, 1997) and libMesh (lib; Kirk *et al.*, 2006). The IBAMR-based LV model was verified in a separate study (Gao *et al.*, 2014b) by comparing quasi-static results of the model under diastolic conditions to a comparable static FE LV model implemented using the commercial ABAQUS FEA (SIMULIA, Providence, RI, USA) software. The IBAMR- and ABAQUS-based models were found to yield good quantitative agreement. We also demonstrated that both models yield good quantitative agreement with earlier experimental and computational results, thereby providing an initial validation of the models; see Gao *et al.* (2014b) for details. The ABAQUS-based version of the LV model is also employed to perform static analyses used to determine the passive material parameters, as described in Section 2.6.

A grid convergence study is performed with Cartesian grid spacings *Δx* = 0.187 cm, *Δx*= 0.156 cm and *Δx* = 0.134 cm with the healthy LV model. Differences in the computed LV cavity volume obtained using the different grid spacings are <3%, and the differences in the computed displacement are <0.05 cm, or 3%, for the entire LV wall at end-diastole and end-systole. Considering the computational efficiency, and our previous validation study (Gao *et al.*, 2014b), which showed that a Cartesian grid spacing of *Δx* = 0.156 cm yielded grid-converged results, we use the grid spacing *Δx* = 0.156 cm for all subsequent simulations.

### 2.6 Material parameter estimation

We determine parameters for the passive and active material models so that the end-diastolic and end-systolic volumes generated by the models are in good agreement with the corresponding volumes derived from the CMR imaging studies.

First, we aim to determine passive parameter material values such that the end-diastolic volume generated by the healthy LV model agrees to within 5% of the end-diastolic volume measured in the CMR study. In this work, these values are empirically determined by a manually directed parameter search that aims to ensure (i) that the isotropic material response is approximately the same as that of Wang *et al.* (2013a), which were obtained from the porcine experimental data of Dokos *et al.* (2002), and (ii) that the relative anisotropic material responses in the fibre, sheet and sheet-normal directions are approximately the same as those of Wang *et al.* (2013a). To decrease the computational requirements of this process, we employ the ABAQUS-based static FE LV model to identify these passive material parameters. For the healthy subject, we identify passive parameters that are somewhat less stiff than those from Wang *et al.* (2013a).

Next, to determine the value of *T*_scale_, which we use to control the strength of contraction, we simulate systolic ejection using the IBAMR-based FSI model with the passive parameters determined by the ABAQUS-based FE model. To do so, we inflate the LV model to the end-diastolic pressure and then initiate systolic contraction, as described in Section 2.4. A value of *T*_scale_ that yields a predicted end-systolic volume that differs from the measured end-systolic volume by <5% is determined by the method of bisection.

In the diseased model, we use the same values of the passive material parameters *a*, *b*, and *b_i_* (*i* = f, s and fs) as are determined for the healthy model, but we modify the anisotropic material response by choosing

(2.25) $$a_{i}^{\mathrm{MI}}=C_{a}^{\mathrm{MI}}\times a_{i}$$

for *i* = f, s and fs. The method of bisection is used to determine a value of $C_{a}^{\mathrm{MI}}$ such that the end-diastolic volume generated by the model under the end-diastolic pressure load agrees with the measured end-diastolic volume to within 5%. As in the healthy model, bisection is again used to determine a value of *T*_scale_ that yields a predicted end-systolic volume that differs from the measured end-systolic volume by <5%.

## 3. Results

### 3.1 Estimated parameters

The model parameters are determined so that the simulated and measured end-diastolic and end-systolic volumes agree to within 5%, as discussed in Section 2.6. Because it is difficult to measure intracardiac pressure *in vivo*, a typical value of 8 mmHg is chosen for the end-diastolic pressure in the healthy model (Wang *et al.*, 2013a), and the end-systolic pressure of 150 mmHg is determined from measurements of the subject's brachial arterial pressure. For the MI patient, who suffers from minor mitral valve regurgitation, we choose a slightly higher end-diastolic pressure of 16 mmHg (Mielniczuk *et al.*, 2007), and as in the healthy model, the end-systolic pressure is estimated from the brachial arterial pressure of the patient, which in this case is 110 mmHg.

For the healthy subject, the measured end-diastolic and end-systolic volumes were determined to be 143 and 61 ml, respectively. Passive material parameters were identified such that the end-diastolic and end-systolic volumes generated by the model are, respectively, 145 and 64 ml, which are both within 5% of the measured values. The empirically determined passive material parameters for the healthy LV are *a* = 0.24 kPa, *b* = 5.08, *a*_f_ = 1.46 kPa, *b*_f_ = 4.15, *a*_s_ = 0.87 kPa, *b*_s_ = 1.6, *a*_fs_ 0.3 kPa and *b*_fs_ 1.3. Using these passive properties, we obtain *T*_scale_ 3.0.

For the patient, the measured end-diastolic and end-systolic volumes were determined to be 116 and 86 ml, respectively, and passive material parameters were determined to yield end-diastolic and end-systolic volumes of 112 and 88 ml, respectively. The scaling factor $C_{a}^{\mathrm{MI}}$ in equation (2.25) is determined to be 7.5. Using these passive properties, we obtain *T*_scale_ 5.5.

**Fig. 5:**
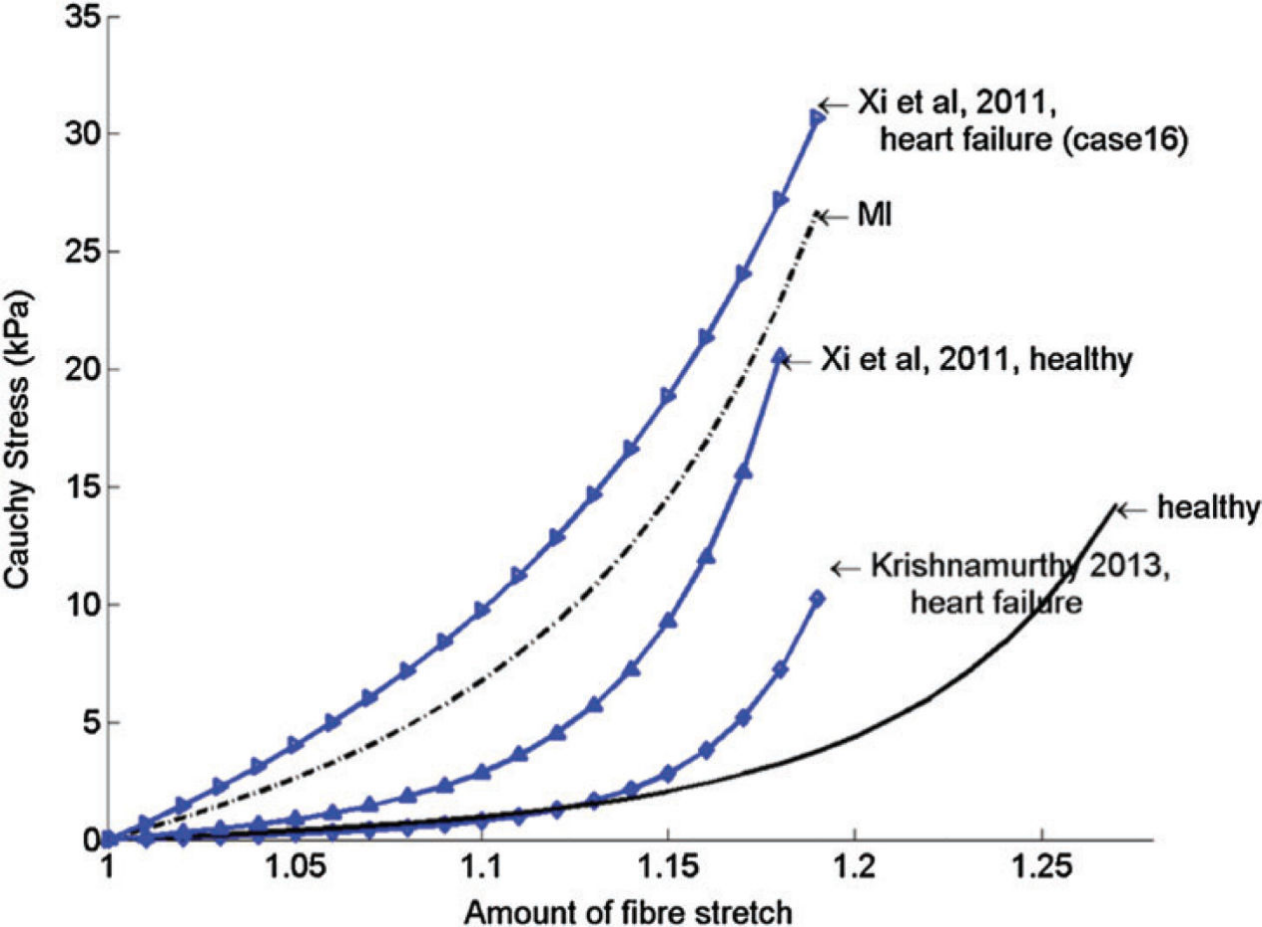
Myofibre stress–strain relationship under uni-axial tension along myofibre direction for the healthy volunteer and the MI patient, compared with results from Xi *et al.* (2011b) and Krishnamurthy *et al.* (2013).

Figure 5 shows the stress–strain relationship for the passive response of the functional myocardium under uni-axial tension in myofibre direction for the models determined for the healthy volunteer and the MI patient, along with corresponding stress–strain relationships obtained in other studies in which myocardial stiffness was also inversely estimated from *in vivo* data (Krishnamurthy *et al.*, 2013; Xi *et al.*, 2011b). Although different constitutive laws and end-diastolic pressure were used, the myofibre stress–strain relationships from our study are comparable with the results of other studies. Note that in the patient model, the passive stiffness of the myocardium is substantially higher than that of the healthy model, even in the unaffected region *U*^un^, which is consistent with results from (Xi *et al.*, 2011b). Likewise, the strength of contraction in the patient is nearly twice that of the subject, despite the lower end-systolic pressure of the patient (110 mmHg compared with 150 mmHg). This larger contractile force is required to overcome the greater passive stiffness of the diseased LV.

### 3.2 Deformation

**Fig. 6:**
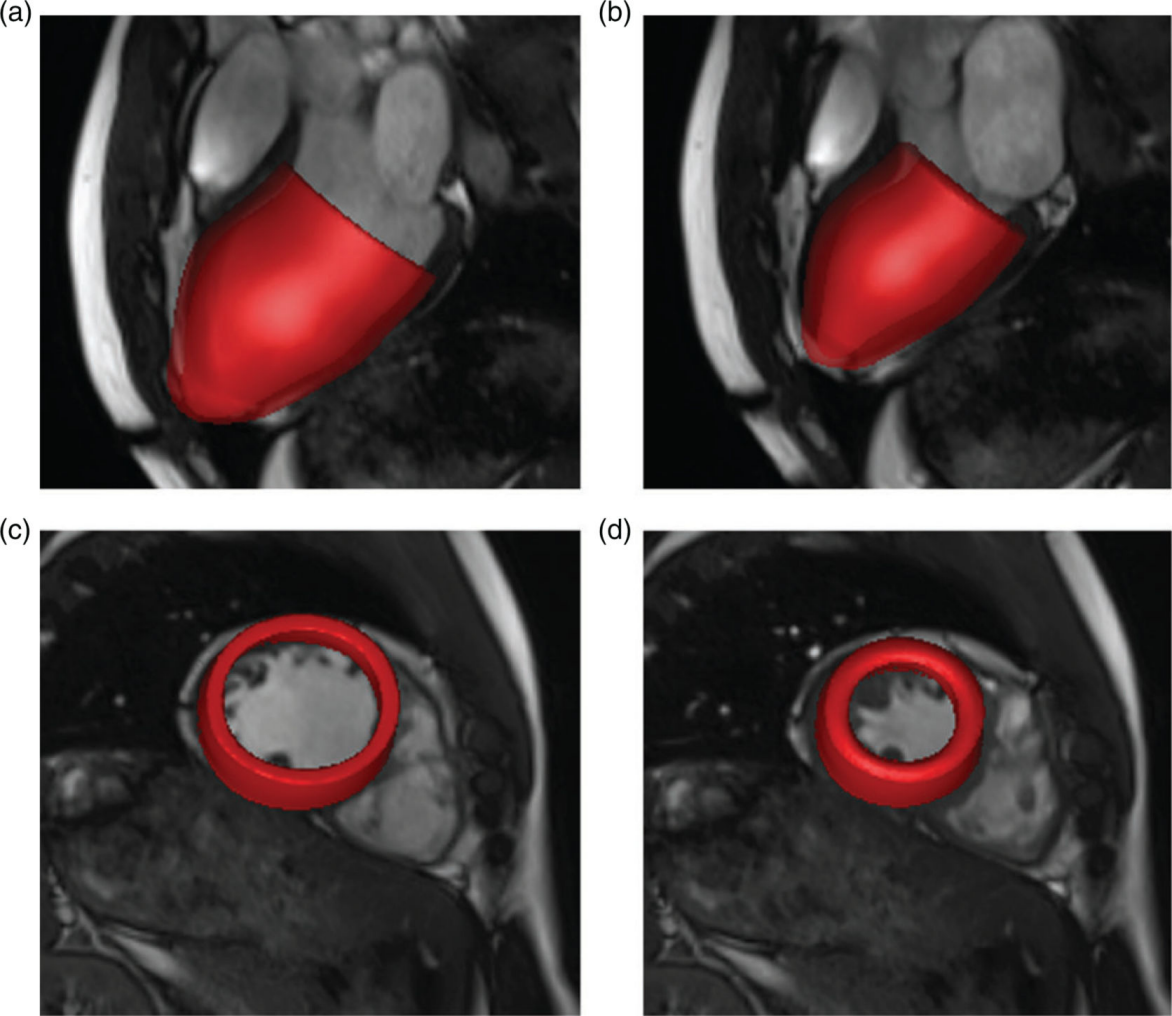
Wall deformations of the healthy LV model at end-diastole (a and c) and end-systole (b and d) superimposed on three-chamber long-axis (a and b) and a short-axis (c and d) views of the CMR cine images.

**Fig. 7:**
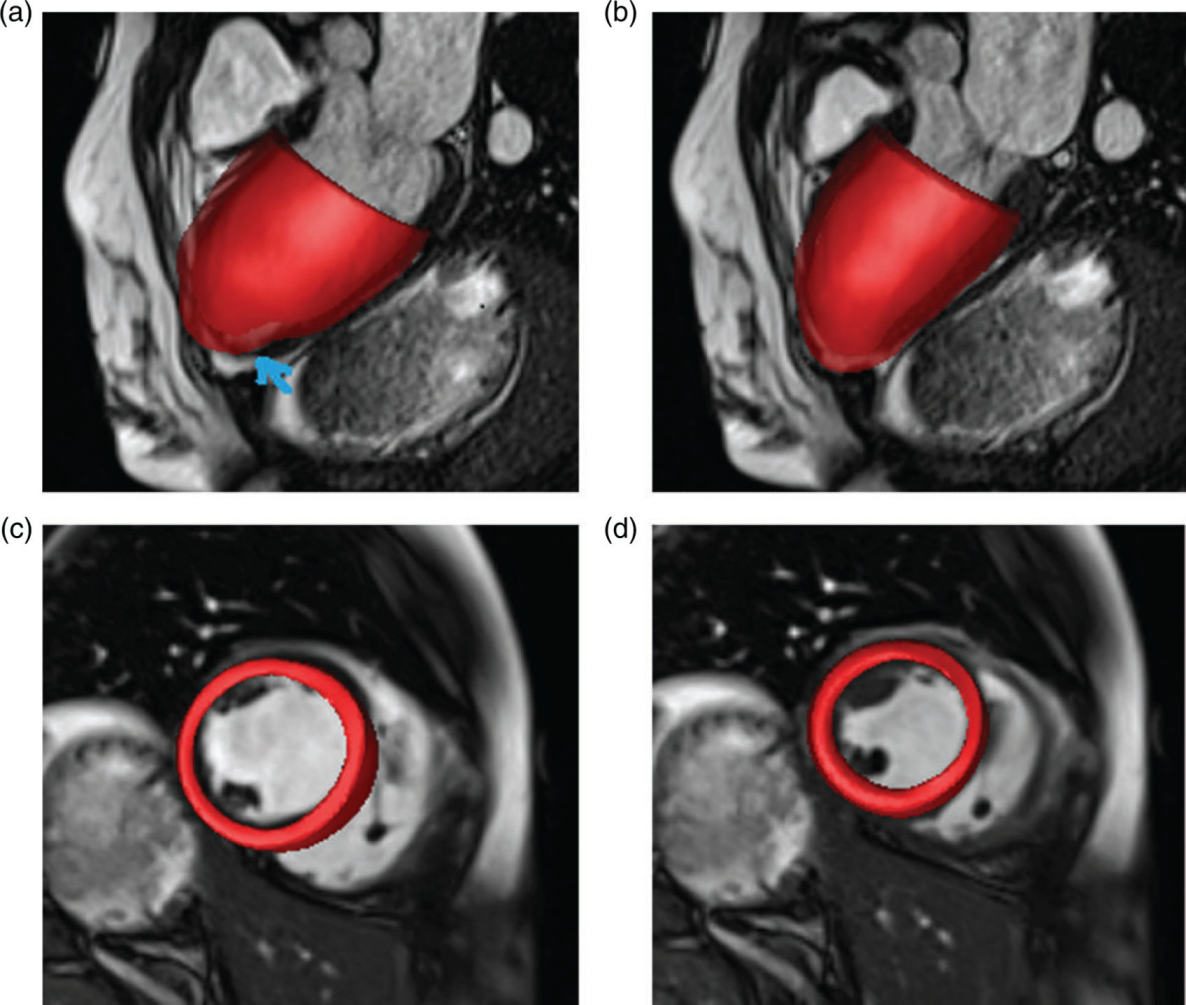
Wall deformations of the diseased LV model at end-diastole (a and c) and end-systole (b and d) superimposed on three-chamber long-axis (a and b) and short-axis (c and d) views of the CMR cine images.

Figure 6 shows the configurations of the healthy LV model at end-diastole and end-systole, respectively, superimposed on long- and short-axis views from CMR cine images of the subject. In general, the deformations of the model are in good agreement with the CMR images, at both end-diastole and endsystole. Small discrepancies can be observed near the apex in the long-axis views; see Fig. 6(a and b). Figure 7 is similar to Fig. 6 but instead shows results from the diseased model and CMR images of the patient. In this case, the discrepancies between the model and the clinical data at the apex are greater, particularly at end-diastole, as indicated by an arrow in Fig. 7(a). Away from the apical regions, however, the simulated endocardial wall deformations of both models agree very well with the CMR measurements.

### 3.3 Active tension

**Fig. 8:**
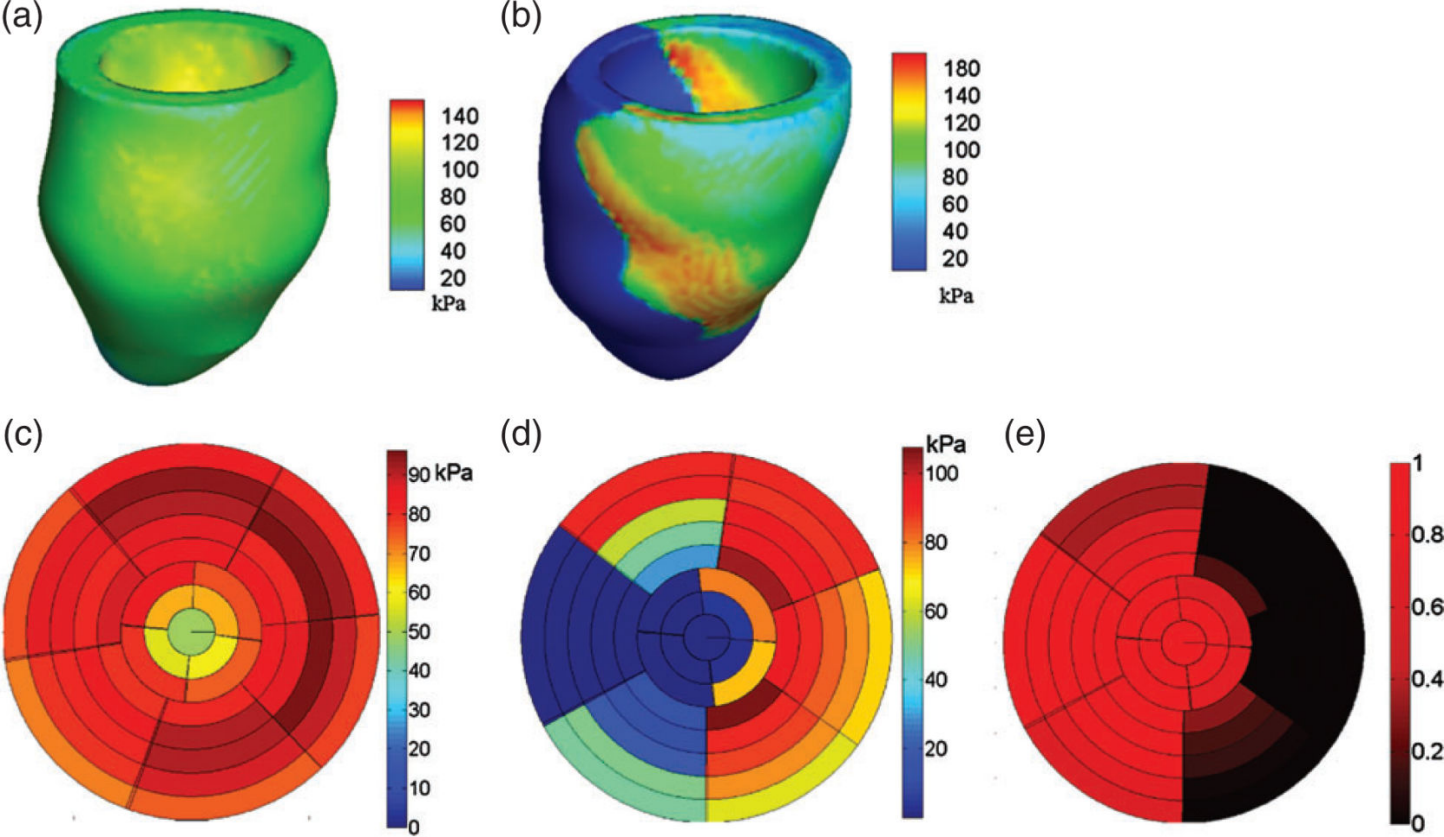
Distributions of active tension *T* at end-systole in the healthy (a) and diseased (b) LV models, regional distributions of *T* from the base to the apex in the healthy (c) and diseased (d) LV models, and regional distribution of MI extent in the diseased LV model (e). The divisions in (d–f) are defined as in Fig. 3. The rings from outer to inner represent the slices from the base to the apex, and each slice is associated with the volumetric region consisting of the points within 5 mm of that slice plane.

Three-dimensional distributions of the active tension *T* computed in both models are shown in Fig. 8(a and b). Using the LV division scheme described in Fig. 3, the end-diastolic active tension in different regions of the LV models is shown in Fig. 8(c and d). In the healthy model, the active tension *T* developed in the LV is quite homogeneous, with a mean value of 81.9 ± 22.6 kPa; see Fig. 8(c). In the diseased model, however, there is substantial heterogeneity. There is no active tension generation in the infarct region, and Fig. 8(b and d) shows that there is a region immediately adjacent to the infarct in which *T* is locally elevated. The average value of *T* in the unaffected healthy myocardium is 86.9 ± 22.7 kPa, which is somewhat higher than that of the healthy LV model.

### 3.4 Stress and strain distributions

**Fig. 9:**
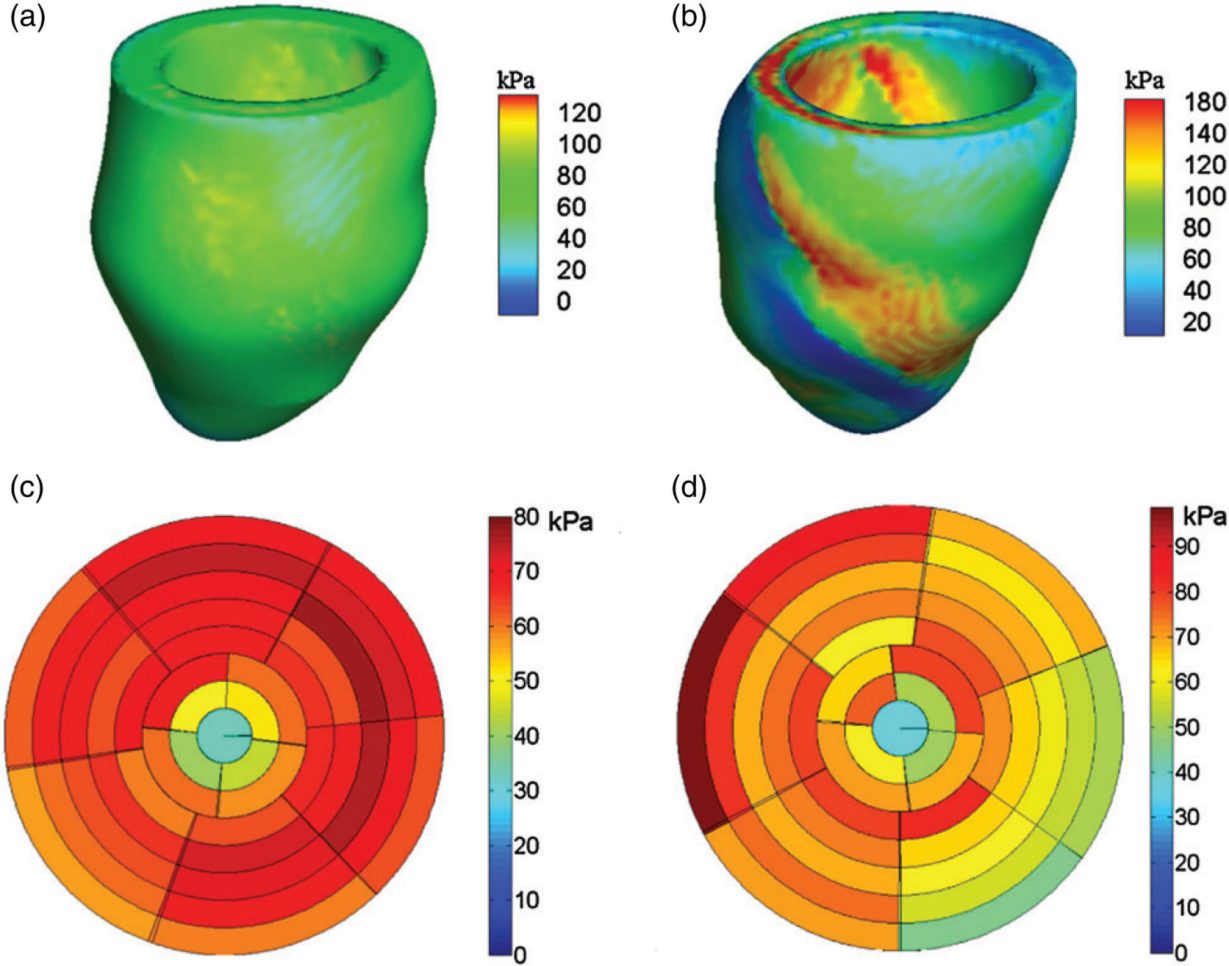
Distributions of fibre stress at end-systole in the healthy (a) and diseased (b) LV models, regional distributions of fibre stress from the base to the apex in the healthy (c) and diseased (d) LV models. Regional distribution of MI extent in the diseased LV model can be found in Fig. 8(e).

Figure 9 shows end-systolic fibre stress distributions for both models. The mean value of the fibre stress is 64 ± 19 kPa in the healthy model. For the diseased LV model, the mean systolic fibre stress is 64 ± 22 kPa in the unaffected myocardium and is 65 ± 32 kPa in the infarct region. We observe an increased passive fibre stress in the region adjacent to the MI. Recall that a similar localized increase in active tension is also observed adjacent to the infarct.

**Fig. 10:**
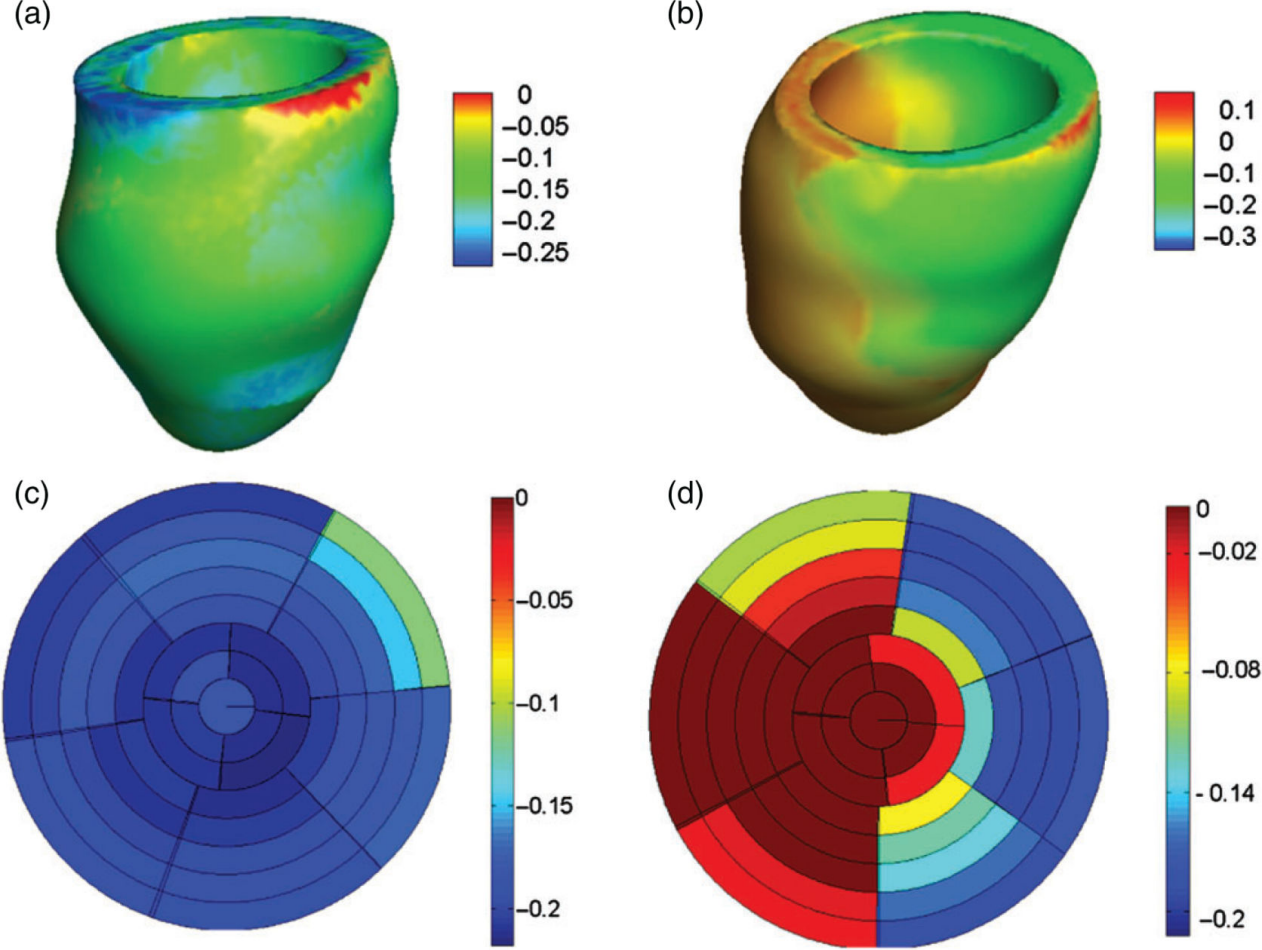
Distributions of fibre strain at end-systole in the healthy (a) and diseased (b) LV models, regional distributions of fibre stress from the base to the apex in the healthy (c) and diseased (d) LV models. Regional distribution of MI extent in the diseased LV model can be found in Fig. 8(e).

Figure 10 shows end-systolic fibre strain distributions for both models. The mean value of the fibre strain is −0.19 ± 0.04 for the healthy model. For the diseased LV model, the mean systolic fibre strain is −0.13 ± 0.06 in the unaffected myocardium and is 0.02 ± 0.02 in the infarct region. Positive systolic fibre strain in the infarct region is a consequence of the ablation of active contraction within that region.

**Fig. 11:**
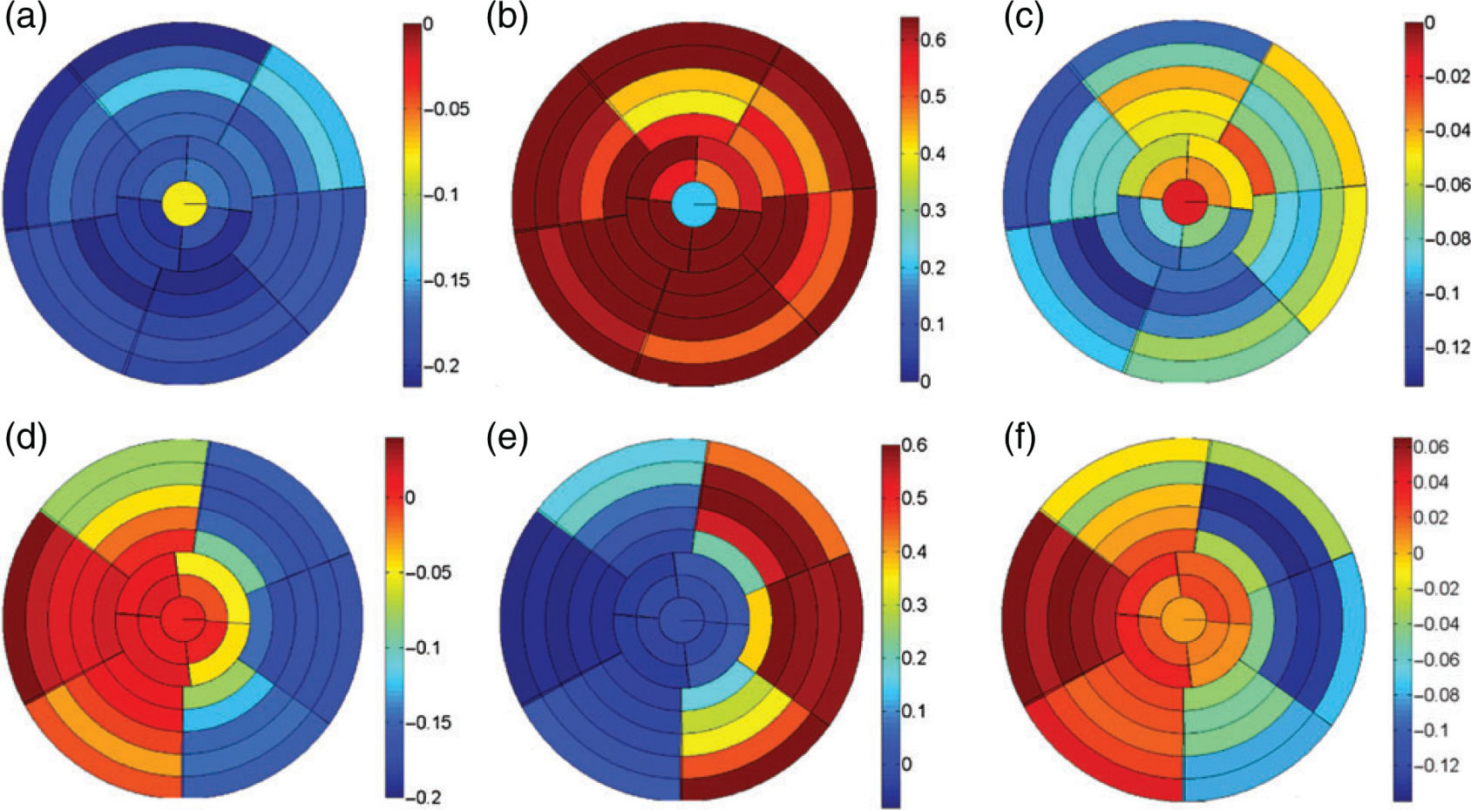
End-systolic circumferential, radial and longitudinal strain distributions in the healthy (a–c) and diseased (d–f) LV models.

Figure 11 plots the distributions of the end-systolic circumferential, radial and longitudinal strains for both models. These results show that for the healthy LV model, the circumferential and longitudinal strains are negative, indicating contraction, with mean values of −0.18 ± 0.05 and −0.07 ± 0.04, respectively, and the radial strains are positive, indicating wall thickening, with a mean value of 0.64 ± 0.25. For the diseased LV model, however, the infarct region experiences positive circumferential strains (0.01 ± 0.02) and longitudinal strains (0.03 ± 0.02) but negative radial strains (−0.04 ± 0.03). The average circumferential, longitudinal and radial strains in the unaffected myocardium are −0.13 ± 0.05, −0.06 ± 0.06 and 0.37 ± 0.23, respectively.

### 3.5 Comparisons of computed and CMR-estimated strains

We compare circumferential strain at end-systole to values obtained from CMR images using a deformable image registration method (Allan *et al.*, 2011). A single mid-ventricular short-axis CMR cine slice is selected for this analysis. Circumferential strain is recovered by tracing myocardial motion from end-diastole to end-systole. A separate study on the accuracy of strain estimation from cine CMR using B-spline deformable image registration was performed that included three healthy volunteers and 41 MI patients (Gao *et al.*, 2014a). This study showed that there is no significant difference between regional systolic circumferential strains obtained from cine CMR and strains determined by DENSE (displacement encoding with stimulated echoes) for both healthy volunteers and patients. Therefore, in this study, only the regional systolic circumferential strain is used for comparison. Table 1 compares regional strains of the computational models to CMR-estimated strains at corresponding locations. Only two regions in the diseased LV, the anterior septum (the central infarct region) and the inferior lateral wall (remote unaffected myocardium), are selected for comparison because it is difficult to trace the complex motions that occur in regions where the myocardium is partially infarcted using the deformable image registration method.

Table 1 shows that the computed and measured systolic strains are generally in good agreement, although there are some discrepancies. In the diseased LV model, the circumferential strain in the anterior septum generated by the model is positive, whereas the values derived from the CMR data are negative. This discrepancy could indicate that some functional myocytes remain within the anterior septal lesion of the patient; such residual active contraction is not considered in the model. Because strain is three-dimensional, there also exist uncertainties in the two-dimensional strains recovered from the deformable registration method that result from through-plane effects.

Table 2 compares average systolic strains generated by the healthy LV model to three-dimensional systolic strains acquired by Moore *et al.* (2000) from normal human hearts. Here, the basal region corresponds to slices 1 and 2, the middle region corresponds to slices 3–5, and the apical region corresponds to slices 6 and 7. It is clear that, except for the longitudinal component, all the strain components are in good agreement with the average values obtained by Moore *et al.* (2000). Specifically, except for the longitudinal strains, all other strains are within one-to-two standard deviations of the clinical data.

### 3.6 LV rotation

**Fig. 12:**
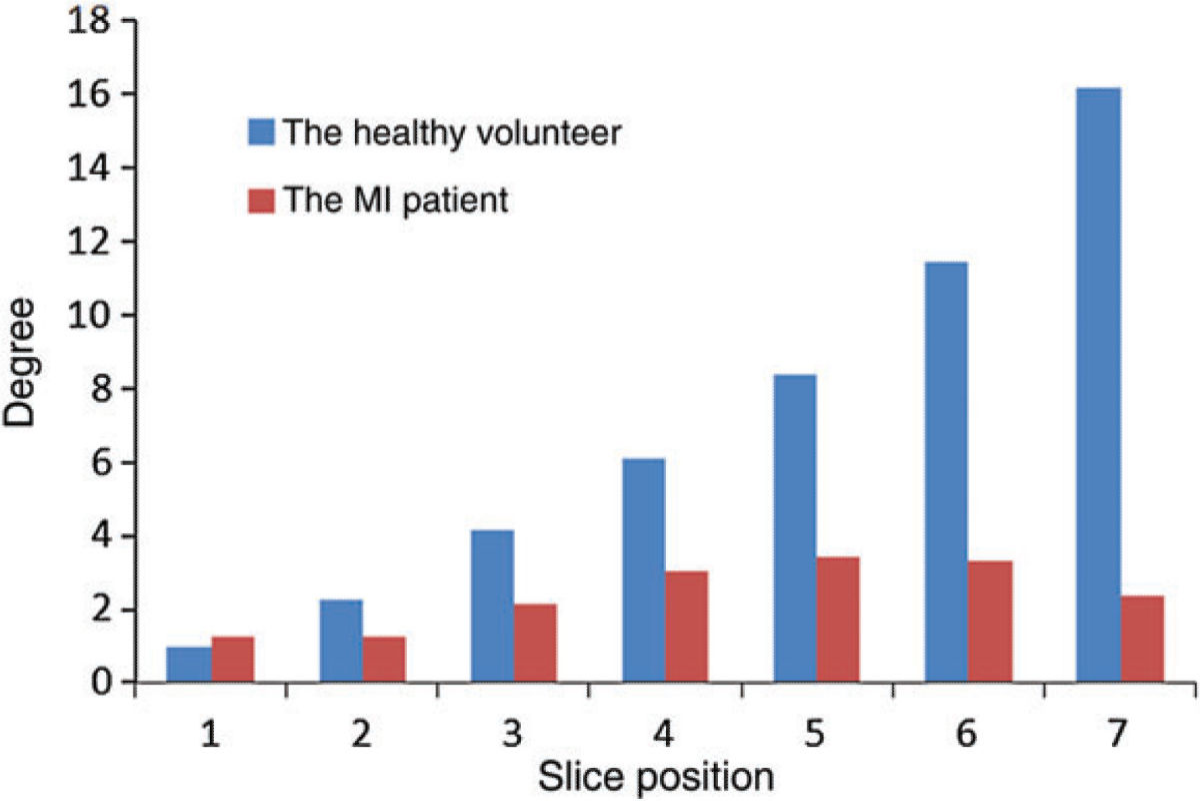
Average rotations along the seven short-axis slices for the healthy and diseased LV models at end-systole. Note that rotations are constrained in both model on the basal plane. The healthy volunteer is shown in blue/left, and the MI patient is shown in red/right.

Figure 12 shows the average rotation of the LV about the long axis from end-systole to end-diastole along the short-axis slices for both the healthy and diseased LV models. In the healthy model, the LV rotation increases approximately linearly from the base to a maximum twist of 16° near the apex. In contrast, the diseased LV model generates very little rotation and achieves a maximum rotation of ~ 3°.

### 3.7 Flow patterns

**Fig. 13:**
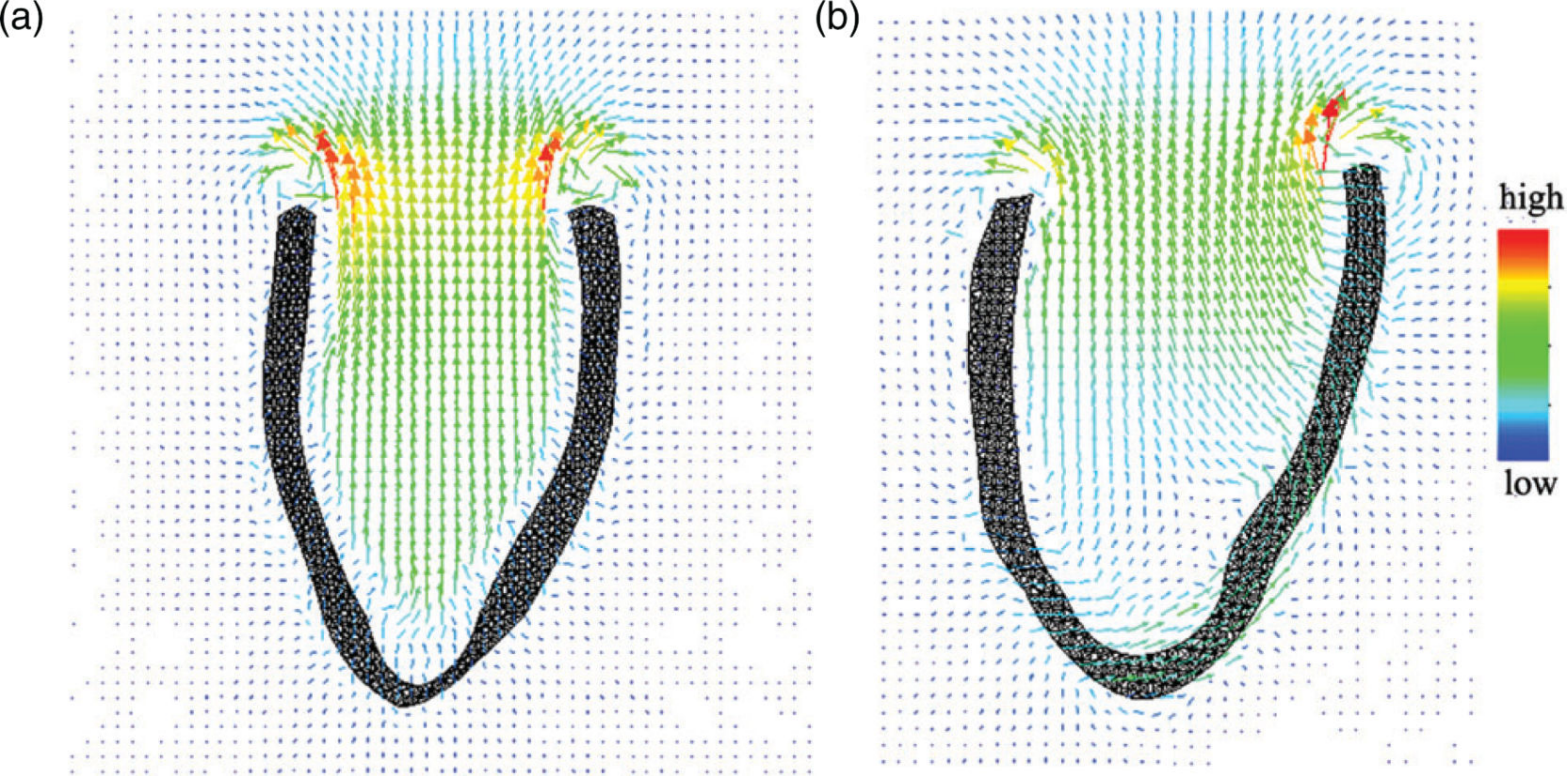
Flow patterns in the healthy (a) and diseased (b) LV models at systole.

The flow patterns at systole are shown in Fig. 13 for both the healthy and the diseased LV model. It is clear that a stronger fluid ejection is provided by the healthy LV model, whereas the diseased LV model generates a much weaker jet of flow, as would be expected given the reduced contractility of the diseased model.

## 4. Discussion

This study presents one of the first IB-based FSI models of ventricular mechanics to incorporate clinical image-based anatomy and regional function along with an orthotropic, invariant-based hyperelastic model of the passive elasticity of the heart (Holzapfel & Ogden, 2009) and a detailed model of cardiac tension generation and excitation–contraction coupling (Niederer *et al.*, 2006). Using this approach, we determined the passive and active material parameters for a healthy LV model based on data acquired from a normal subject, and for a diseased LV model based on data from a patient 6-months after an acute MI. Using these parameterizations, we quantified regional stress and strain distributions, compared the model results to clinical strain data and simulated the dynamics of active contraction and the resulting intracardiac blood flow.

Since its introduction, the IB method has been used extensively to model the dynamics of the heart and its valves (Peskin & McQueen, 1996; McQueen & Peskin, 1997, 2000; Kovács *et al.*, 2001; McQueen & Peskin, 2001; Griffith *et al.*, 2007, 2009a,b; Griffith, 2012; Luo *et al.*, 2012; Ma *et al.*, 2013), but the IB models described here are among the first to use detailed descriptions of the passive elastic response and active sarcomeric force generation of the ventricular myocardium. Most previous IB models of the heart use descriptions of the ventricular myocardium in which the heart muscle is modelled using discrete collections of elastic fibres rather than a continuum mechanics-type approach; see (McQueen *et al.*). Although such ‘explicit fibre’ models are well suited for describing extremely anisotropic materials, it is challenging to link such descriptions to experimental data, or to incorporate details of the material shear response. Further, although constructing explicit elastic fibre-based models from clinical imaging data is feasible (McQueen *et al.*), at present, doing so is time consuming and requires specialized software tools. The IB method is not restricted to fibre-based elasticity models, however, and recent extensions of the IB framework, including that used herein (Griffith & Luo), have enabled the use of finite-strain elasticity models within the IB framework (Zhang *et al.*, 2004; Liu *et al.*, 2006; Zhang & Gay, 2007; Boffi *et al.*, 2008; Heltai & Costanzo, 2012; Griffith & Luo).

Our use of the IB method to describe the dynamics of the LV enables us to account for the inertia of the LV wall as well as the blood. However, one of the key limitations of the present models is that they lack descriptions of any structures above the base of the heart, including the valve leaflets and valve rings. Therefore, although our simulation framework is capable of describing FSI, the flow patterns generated by the present models are not physiologically accurate; therefore we focus on analysing the structural deformations, strains and stresses of the LV model. Adapting this simplified LV geometry is the first step towards a more complete description of the heart, and it facilitates qualitative comparisons between the present model and previously published LV models, almost all of which are simplified in a similar manner (Krishnamurthy *et al.*, 2013; Niederer *et al.*, 2011a; Wang *et al.*, 2013a,b; Wenk *et al.*, 2011a,b, 2012; Xi *et al.*, 2011b). Indeed, we are actively working to incorporate such models into our LV simulation framework. We remark that even with our relatively simple models of cardiac FSI, we are already able to obtain insight into the flow behaviour during systolic contraction. In the healthy LV model, the contracting myocardium imparts substantial momentum to the blood in the entire cavity, resulting in substantial fluid motion from the apex to the base. In contrast, the blood is nearly stagnant in the apical region of the diseased LV model, which could increase the thrombogenic potential or even lead to further adverse remodelling.

In our simulations, we find that for the healthy LV model, fibre strain is relatively uniform during contraction because of the underlying myocardial structure, which suggests efficient LV contraction across the whole wall (Costa *et al.*, 1999; Rijcken *et al.*, 1999; Tseng *et al.*, 2000). Systolic fibre strain and active contractile force are likewise almost homogeneous in the healthy model. In contrast, distributions of fibre stress and strain and active tension in the MI model show substantial variation and regional heterogeneity, as also demonstrated by Guccione *et al.* (1995). CMR studies also have demonstrated that systolic strains in infarcted myocardial tissue are reduced substantially *in vivo* (Antoni *et al.*, 2010). In the diseased LV model, a region of high-fibre stress adjacent to the MI also develops, as in Fig. 9(a and c), and this band of increased fibre stress has been suggested as a possible cause of further adverse remodelling (Grossman, 1980; Pfeffer & Braunwald, 1990). Systolic LV rotation, which is caused by the contractions of the helically arranged myofibres, is also considered to be an important clinical index of global cardiac performance (Lamia *et al.*, 2011). The healthy LV model predicts a maximum systolic rotation of 16° in the apical region, but this is reduced to 3° in the diseased LV. These values are within the range reported from *in vivo* measurements (Fuchs *et al.*, 2004). Recall, however, that rotations of the basal plane are constrained in our models, and so we are unable to capture the substantial clockwise basal rotation of the real heart.

We observe some differences between the computed and measured LV deformations, particularly near the apex, as shown in Figs. 6 and 7. There are several possible causes of these discrepancies. First, in the healthy model, we used homogeneous material parameters, but the material parameters for the apical region might be greatly different from other regions (Nordsletten *et al.*, 2010). In the diseased model, we employ heterogeneous material parameters, but these parameters are still homogeneous within the unaffected and infarcted regions and likely do not account for the true heterogeneity of the tissue. Further, in our model, the MI region is considered to be totally non-contractile, but essentially akinetic regions may still include a subpopulation of viable, contractile myocytes, even for chronic MIs (Walker *et al.*, 2007), and the strength of contraction should vary in the MI region depending on the severity of infarction, as suggested by Hoffer *et al.* (1999). Our model also does not include a description of the pericardium, which is much stiffer than the myocardium and which is tethered near the apical region of the heart. Finally, longitudinal and circumferential displacements of the basal plane of the LV are approximately fixed in our models, similar to the simplified boundary conditions used by Wenk *et al.* (2011a). In future work, the models can be improved by applying measured basal motions (Xi *et al.*, 2011b) once detailed basal measurement from CMR becomes available in our clinical imaging facilities. Alternatively, a more complete heart model with descriptions of the inflow and outflow tracts would be expected to produce more realistic basal deformations, and we are presently working to develop such models.

Estimating material parameters from measured data that may be reasonably obtained in a clinical setting remains a significant challenge for subject-specific modelling. In this study, we used pressure–volume relations to characterize the passive and active material parameters because CMR strain data were not available. Our estimated passive myocardial parameters are substantially less stiff than those determined in earlier studies (Holzapfel & Ogden, 2009; Wang *et al.*, 2009), which were fit to experimental shear response data acquired from *ex vivo* porcine tissue. We also determined that recovering the clinically measured end-diastolic and end-systolic volumes required us to employ substantially different material parameters in the healthy and diseased LV models, and we expect further differences would be observed for additional subjects.

In a separate study on the passive material parameter estimation (Gao *et al.*), we found that although individual parameters cannot be uniquely defined due to measurement errors and correlations between parameters, the mechanical response in terms of the myofibre stress–strain relation determined using our approach is quite robust. Indeed, our estimated myofibre stress–strain for functional myocardium in the healthy volunteer and the MI patient is comparable with other studies, as shown in Fig. 5. In particular, the stiffness recovered for the MI patient-derived model is in good agreement with the stiffness reported for a heart failure patient by Xi *et al.* (2011b).

In the systolic phase, the active stress generated by the myocytes is rescaled by the parameter *T*_scale_, whereas the remaining parameters of the active contraction model are kept fixed. This allows us to match the measured LV dynamics in end-systole for both LV models. The same approach is used to avoid the complexity of having to determine many different parameters from the electromechanical models. This approach has been widely used by other groups (Sun *et al.*, 2009; Wenk *et al.*, 2011a, 2012; Krishnamurthy *et al.*, 2013; Wang *et al.*, 2013b). The optimized maximum isometric tension at the resting sarcomere length is 168.6 kPa in the healthy volunteer, which is much lower than the value from the MI patient, which is 309.1 kPa. Similar to our study, Wang *et al.* (2013b) reported much higher isometric tension in non-ischaemic heart failure patients compared with healthy volunteers. Studies from sheep heart modelling with chronic MI have reported maximum isometric tension in a range of 180 kPa (Wenk *et al.*, 2011b) to 500 kPa (Wenk *et al.*, 2011a) at the maximum sarcomere length. In our active contraction model, the maximum isometric tension at maximum sarcomere length is ~536 kPa for the functional myocytes from the MI patient and 296 kPa for the healthy volunteer.

Wenk *et al.* (2011a) determined that the stiffness in the MI regions was 50 times greater than functional myocardium. They also concluded that it was possible that the true ratio could be higher than the usually used value (10 times) (Sun *et al.*, 2009). Indeed, for our diseased LV model, we found that it was necessary to increase the stiffness within the infarction by a factor of 50 when compared with the passive stiffness of the distal unaffected tissue in order to match measured end-systolic volume. A transition region (border zone) is defined between the infarcted region and the functional myocardium to avoid the abrupt change of material properties. This approach was also used by Nordsletten *et al.* (2010), who defined a similar transition region within a distance of ~10% of the long axis of the LV. However, the discrepancy between the disagreements in the predicted and measured deformations, as shown in Fig. 7(a), suggests that this approach only gives a simplified model for the scar. To accurately define the border zone and the material properties within and around the scar will require the development of new imaging protocols and detailed experimental tests.

Other limitations include the fact that we have used only a simplified model of tissue excitation that assumes spatially uniform calcium dynamics, and that the fibre architectures of our models are rule-based. We are presently working to incorporate descriptions of excitation propagation in our models, which will yield more realistic activation patterns. To acquire realistic myocardial fibre angle distributions throughout the LV wall, it is possible to use diffusion tensor MRI (DT-MRI). At present, however, DT-MRI yields high-resolution fibre data only in *ex vivo* hearts (Tseng *et al.*, 2002), and although it is possible to use DT-MRI to obtain *in vivo* fibre data (Toussaint *et al.*, 2010), resolution is relatively low. In general, rule-based myofibre generation procedures have been found to provide a reasonable approximation of the true fibre structure of the heart (Bishop *et al.*, 2009). The use of such models, however, may contribute to the discrepancy between the computed systolic longitudinal strains and the clinical data as reported by Gil *et al.* (2012) (see Table 2). Specifically, they may lead to underestimating the long-axis shortening during systolic contraction.

We remark that knowledge of the unstressed configuration of the LV is important when modelling the elastic response of the heart; however, the heart is continuously loaded *in vivo*. Moreover, even if all *in vivo* loads are removed, the LV wall possesses residual stresses that are the consequence of tissue growth and remodelling. Consequently, it is extremely difficult to determine the unstressed configuration directly from clinical images. Earlier studies have used the LV geometry in early-to-mid diastole as the unloaded and unstressed reference state, as we have done here, because that configuration experiences minimal endocardial pressure loading (Sun *et al.*, 2009; Sermesant & Razavi, 2010; Wenk *et al.*, 2012). Nordsletten *et al.* (2010) estimated the unloaded LV geometry via an empirical formula proposed by Klotz *et al.* (2006), and we and others have recovered the unloaded LV geometry at end-diastole using a multiplicative decomposition of the deformation gradient tensor or a backward displacement method (Aguado-Sierra *et al.*, 2011; Wang *et al.*, 2014). It is also possible to incorporate residual stresses by modifying the constitutive model used to describe the passive response of the tissue, as we have done in an earlier study (Wang *et al.*, 2014). We do not include residual stresses in the present work because of the lack of knowledge of subject-specific residual strains. Further, because different tissue constituents likely have different natural or stress-free configurations, incorporating residual stresses within such models presents both theoretical and practical challenges that merit further investigation.

Finally, it is worth mentioning that there is a clear need for carefully designed validation studies before these models may be successfully applied in clinical practice. Although clinical imaging is able to provide cardiac deformation and flow fields, *in vivo* strains are still somewhat limited and the direct imaging of stress is not presently feasible. Moreover validating the predictions of the models of active tension generation at the cellular level is extremely difficult, although measurement of active tension in ‘skinned’ animal LV myofibres is feasible (Sun *et al.*, 2009). Therefore, this study focuses on LV biomechanical behaviours at the tissue level. Follow-up studies involving large cohorts of healthy subjects and patients will be needed to achieve clinically applicable models.

## 5. Conclusions

In this study, we have developed dynamic descriptions of human left ventricular biomechanics in health and disease. Our models incorporate an invariant-based hyperelastic model of the passive elastic response of the LV and a detailed description of excitation–contraction coupling and active force generation, as well as the inertia of the ventricular muscle and the blood, based on a FE-IB framework. Cardiac anatomy and regional function are derived from CMR imaging studies of a healthy subject and a patient 6 months after an acute MI. The material parameters of the healthy and diseased models are determined from these clinical data to capture pressure–volume relationships as well as strains derived from the CMR imaging studies.

Despite simplifications introduced in these models, they predict detailed displacement and strain distributions that are generally in good agreement both with our own CMR measurements and also with earlier clinical studies. We found that the diseased model yields much lower strains and rotations during contraction compared with the healthy model. Hence, contraction power is greatly reduced by the presence of the scared tissue. This loss of contractility is also clearly reflected in the flow patterns at systole, in which jet-like systolic ejection is greatly reduced in the diseased model. This personalized modelling approach enables us to predict in great detail the functional changes of the left ventricle post-MI, and could possibly lead to the development of improved approaches to patient risk stratification and treatment planning.

## Figures and Tables

**Table 1:** Comparison between the predicted and CMR-estimated systolic circumferential strains in the mid LV

healthy LV	infsept	antsept	anterior	antlat	inflat	inferior
IB/FE	–0.19	–0.17	–0.14	–0.16	–0.18	–0.19
CMR	–0.16	–0.15	–0.15	–0.14	–0.18	–0.11
diseased LV		antsept			inflat	

IB/FE	–	0.02	–	–	–0.17	–
CMR	–	–0.05	–	–	–0.20	–

**Table 2:** Systolic strains in the healthy model in different LV regions

	Septal			Lateral		
Strain	Inferior septal	Anterior septal	Anterior	Anterior lateral	Inferior lateral	Inferior
*E*_cc_						
Basal	-0.18 ± 0.04^*^	-0.20 ± 0.05^*^	-0.18 ± 0.05^*^	-0.14 ± 0.03	-0.18 ± 0.03^*^	-0.18 ± 0.03^*^
Middle	-0.20 ± 0.05^**^	-0.18 ± 0.05^*^	-0.16 ± 0.03^**^	-0.17 ± 0.03^**^	-0.18 ± 0.03^**^	-0.20 ± 0.04^*^
Apical	-0.20 ± 0.05^*^		-0.17 ± 0.05^**^	-0.17 ± 0.05^**^		-0.20 ± 0.06^*^
*E*_rr_						
Basal	0.70 ± 0.20	0.87 ± 0.20	0.87 ± 0.21	0.73 ± 0.23^**^	0.64 ± 0.22^*^	0.63 ± 0.24^**^
Middle	0.75 ± 0.24^**^	0.58 ± 0.20^*^	0.45 ± 0.13^*^	0.50 ± 0.13^*^	0.67 ± 0.19^**^	0.82 ± 0.20
Apical	0.84 ± 0.25		0.63 ± 0.20^*^	0.55 ± 0.22^*^		0.80 ± 0.30^**^
*E*_ll_						
Basal	-0.1 ± 0.03^*^	-0.12 ± 0.04^*^	-0.09 ± 0.04^**^	-0.06 ± 0.03	-0.06 ± 0.03	-0.07 ± 0.03
Middle	-0.12 ± 0.02^*^	-0.08 ± 0.03	-0.05 ± 0.03	-0.06 ± 0.03^**^	-0.08 ± 0.02^**^	-0.10 ± 0.02^**^
Apical	-0.10 ± 0.03^**^		-0.05 ± 0.03	-0.04 ± 0.02		-0.09 ± 0.03
*E*_cr_						
Basal	-0.15 ± 0.09	-0.18 ± 0.07	-0.25 ± 0.06	-0.30 ± 0.09	-0.18 ± 0.13^**^	-0.13 ± 0.13^**^
Middle	0.10 ± 0.08^*^	0.007 ± 0.14^*^	0.02 ± 0.15^**^	0.08 ± 0.12^*^	0.18 ± 0.07	0.20 ± 0.05
Apical	0.17 ± 0.06^*^		0.17 ± 0.04^*^	0.11 ± 0.07^*^		0.15 ± 0.08^**^
*E*_cl_						
Basal	-0.03 ± 0.03^*^	-0.03 ± 0.03^*^	-0.01 ± 0.03^*^	0.01 ± 0.03^*^	-0.004 ± 0.05^*^	-0.03 ± 0.04^**^
Middle	-0.03 ± 0.02^**^	-0.02 ± 0.03^*^	-0.01 ± 0.04^**^	-0.01 ± 0.03^*^	-0.02 ± 0.03^**^	-0.03 ± 0.02^**^
Apical	-0.03 ± 0.02^**^		-0.02 ± 0.02^**^	-0.03 ± 0.02		-0.04 ± 0.02^**^
*E*_rl_						
Basal	0.01 ± 0.18^*^	-0.03 ± 0.17^*^	-0.05 ± 0.16^*^	-0.07 ± 0.17^**^	-0.05 ± 0.16^**^	0.04 ± 0.16^*^
Middle	0.01 ± 0.06^**^	-0.07 ± 0.07	-0.04 ± 0.07^*^	0.06 ± 0.06^*^	0.06 ± 0.06^*^	0.06 ± 0.04^*^
Apical	-0.05 ± 0.11^**^		-0.05 ± 0.08^*^	-0.002 ± 0.07^*^		-0.005 ± 0.09^*^

The strain components are *E*_cc_, circumferential; *E*_rr_, radial; *E*_ll_, longitudinal; *E*_cr_, circumferential-radial shear; *E*_cl_, circumferential-longitudinal shear and *E*_rl_, radial-longitudinal shear.

* indicates that the mean value lies in with one standard deviation (two standard deviations) of the corresponding data in Moore *et al.* (2000).

** indicates that the mean value lies in with one standard deviation (two standard deviations) of the corresponding data in Moore *et al.* (2000).

## Appendix A. Active tension generation

In the sarcomere, contractile force is generated by cross-bridge cycling between the thin actin filaments and thick myosin filaments. When the cell is at rest, myosin-binding sites along the actin filaments are inaccessible because they are covered by tropomyosin, a highly extended protein situated in the actin groove. These myosin binding sites are revealed by the action of troponin, a protein associated with actin and tropomyosin. When bound to calcium, troponin undergoes a conformational change that acts to shift tropomyosin and thereby to allow actin-myosin cross-bridge cycling and active tension generation. High levels of intracellular calcium are stimulated by the electrical activation of the cells, in which inward transmembrane calcium currents induce the release of calcium stores in the sarcoplasmic reticulum (calcium-induced calcium release). For details on the physiology of active contraction and excitation–contraction coupling, see the monograph of Bers (2001).

In our models, the intracellular calcium concentration [Ca]_i_ is taken to be spatially uniform and to satisfy the dynamics

(A.1) $$\frac{d{\left[\mathrm{Ca}\right]}_{i}}{dt}=\frac{{\left[\mathrm{Ca}\right]}_{i,\max}-{\left[\mathrm{Ca}\right]}_{i,0}}{\tau_{\mathrm{Ca}}}\exp^{\left(1-t∕\tau_{\mathrm{Ca}}\right)}\left(1-\frac{t}{\tau_{\mathrm{Ca}}}\right),$$

in which [Ca]_i,max_ = 1.0 μM, [Ca]_i,0_ = 0.01 μM, and *τ*_Ca_ 0.06 s.

In our simulations, active tension development in the sarcomeres is governed by the model of Niederer *et al.* (2006), which describes the dynamics of active tension development as a function of [Ca]_i_ and the fibre stretch *λ*_f_ and the time rate of change of fibre stretch. In this model, the dynamics of the concentration of troponin-bound calcium [Ca]_trpn_ are described by

(A.2) $$\frac{d{\left[\mathrm{Ca}\right]}_{\mathrm{trpn}}}{dt}=k_{\mathrm{on}}{\left[\mathrm{Ca}\right]}_{i}\left({\left[\mathrm{Ca}\right]}_{\mathrm{trpn},\max}-{\left[\mathrm{Ca}\right]}_{\mathrm{trpn}}\right)-k_{\mathrm{off}}{\left[\mathrm{Ca}\right]}_{\mathrm{trpn}}$$

(A.3) $$k_{\mathrm{off}}=k_{\mathrm{off},\mathrm{ref}}\left(1-\frac{T}{\gamma T_{\mathrm{ref}}}\right),$$

in which *k*_on_ is the binding rate, *k*_off_ is the unbinding rate, *k*_off,ref_ is the unbinding rate in the absence of active tension, *T* is the active tension and *T*_ref_ is the maximum tension at the resting sarcomere length. The fraction of actin binding sites *z* available for cross-bridge cycling is governed by

(A.4) $$\frac{dz}{dt}=\alpha_{0}{\left(\frac{{\left[\mathrm{Ca}\right]}_{\mathrm{trpn}}}{{\left[\mathrm{Ca}\right]}_{\mathrm{trpn},50}}\right)}^{n}\left(1-z\right)-\alpha_{r1}z-\alpha_{r2}\frac{z^{n_{r}}}{z^{n_{r}}+K_{z}^{n_{r}}},$$

in which [Ca]_trpn,50_ is the concentration of troponin-bound calcium at half-activation,

(A.5) $${\left[\mathrm{Ca}\right]}_{\mathrm{trpn},50}={\left[\mathrm{Ca}\right]}_{\mathrm{trpn},\max}{\left[\mathrm{Ca}\right]}_{50}∕\left({\left[\mathrm{Ca}\right]}_{50}+\frac{k_{\mathrm{off},\mathrm{ref}}}{k_{\mathrm{on}}}\left(1-\frac{1+\beta_{0}\left(\lambda_{f}-1\right)}{2\gamma }\right)\right),$$

with [Ca]_50_ = [Ca]_50,ref_(1 + *β*_1_(*λ*_f_ – 1)). The isometric tension is defined by

(A.6) $$T_{0}=T_{\mathrm{ref}}\left(1+\beta_{0}\left(\lambda_{f}-1\right)\right)\frac{z}{z_{\max}},$$

in which *λ*_f_ is stretch in the fibre direction, and *z*_max_ is determined by

(A.7) $$z_{\max}=\left(\frac{\alpha_{0}}{{\left({\left[\mathrm{Ca}\right]}_{\mathrm{trpn},50}∕{\left[\mathrm{Ca}\right]}_{\mathrm{trpn},\max}\right)}^{n}}-K_{2}\right)/\left(\alpha_{r1}+K_{1}+\frac{\alpha_{0}}{{\left({\left[\mathrm{Ca}\right]}_{\mathrm{trpn},50}∕{\left[\mathrm{Ca}\right]}_{\mathrm{trpn},\max}\right)}^{n}}\right),$$

with

(A.8) $$K_{1}=\frac{\alpha_{r2}z_{p}^{n_{r}-1}n_{r}K_{z}^{n_{r}}}{{\left(z_{p}^{n_{r}}+K_{z}^{n_{r}}\right)}^{2}},$$

(A.9) $$K_{2}=\alpha_{r2}\frac{z_{p}^{n_{r}}}{z_{p}^{n_{r}}+K_{z}^{n_{r}}}\left(1-\frac{n_{r}K_{z}^{n_{r}}}{z_{p}^{n_{r}}+K_{z}^{n_{r}}}\right).$$

The active tension *T* is calculated from a fading memory model,

(A.10) T=T0×{1+a∑i=13Qi1−∑i=13Qiif∑i=13Qi<0,1+(2+a)∑i=13Qi1+∑i=13Qiotherwise,}

in which *Q_i_* (*i* = 1, 2, 3) are determined by

(A.11) $$\frac{dQ_{i}}{dt}=A_{i}\frac{d\lambda_{f}}{dt}-\alpha_{i}Q_{i}.$$

Following Niederer *et al.* (2006), the model parameters are *a* = 0.35, *A*_1_ = −29, *A*_2_ = 138, *A*_3_ = 129, *α*_0_ = 8 s^−1^, *α*_1_ = 30 s^−1^, *α*_2_ = 130 s^−1^, *α*_3_ = 625 s^−1^, *α*_r1_ = 2.0 s^−1^, *α*_r2_ = 1.75 s^−1^, *β*_0_ = 4.9, *β*_1_ −4.0, [Ca]_50,ref_ = 1.05 μM, [Ca]_trpn,max_ = 70 μM, *γ* = 2.0 *k*_on_ = 100 μM^−1^ s^−1^, *k*_off,ref_ = 200 s*−1*, *K*_z_ = 0.15, *n* = 3, *n*_r_ = 3 and *z*_p_ = 0.85. We use *T*_ref_ = *T*_scale_ × 56.2 kPa, in which the value of the dimensionless scale factor *T*_scale_ is determined by matching the end-systolic volume of the model LV to the *in vivo* LV volume determined non-invasively via CMR imaging. The value of *T*_ref_ with *T*_scale_ = 1 corresponds to that used by Niederer *et al.* (2006).
